# Distinct Phage‐Encoded Enzymes for Substitution of Deoxythymidine by Deoxyuridine in Phage Genomes

**DOI:** 10.1002/advs.202512937

**Published:** 2025-09-26

**Authors:** Yating Li, Jason Tan, Yanqin Tu, Jingnan Wu, Zaifang Zhang, Yifeng Wei, Xinan Jiao, Yan Zhou

**Affiliations:** ^1^ Jiangsu Key Laboratory of Zoonosis Yangzhou University Yangzhou 225009 China; ^2^ Key Laboratory of Prevention and Control of Biological Hazard Factors (Animal Origin) for Agrifood Safety and Quality Ministry of Agriculture of China Yangzhou University Yangzhou 225009 China; ^3^ Jiangsu Co‐Innovation Center for Prevention and Control of Important Animal Infectious Diseases and Zoonoses Yangzhou University Yangzhou Jiangsu Province 225009 China; ^4^ Singapore Institute of Food and Biotechnology Innovation (SIFBI) Agency for Science Technology and Research (A*STAR) Singapore 138669 Singapore; ^5^ College of Bioscience and Biotechnology Yangzhou University Yangzhou Jiangsu Province 225009 China

**Keywords:** bacteriophages, biosynthetic pathways, dCTP deaminases, dTMP phosphatased, DTTP pyrophosphatases, dU‐DNA, restriction endonucleases

## Abstract

DNA base modification is a common strategy used by bacteriophages to evade host immune detection. A prominent example is dU‐DNA, where thymidine is globally replaced with 2′‐deoxyuridine. Despite its widespread occurrence, dU‐DNA's biosynthetic pathways and functional roles remain incompletely understood. Here, different enzymes supporting dU‐DNA biosynthesis in phage PBS1, Roseophage DSS3_VP1, and Yersiniophage PhiR1‐37 are identified and characterized. The dU nucleotide precursor is supplied by phage dCTP deaminases (Dcds). Thymidine nucleotides are degraded by dTMP phosphatases (Dtms) in PBS1 and DSS3_VP1, and by a dTTP pyrophosphatase (Dtt) in PhiR1‐37, preventing incorporation into phage DNA. The dU‐DNA, isolated from Yersiniophage PhiR1‐37 or synthesized by PCR, demonstrates resistance to cleavage by restriction enzymes recognizing thymidine‐containing sequences, partial resistance to *Lb*Cas12a nuclease recognizing a TTTV PAM site, while remaining sensitive to *Sp*Cas9 nuclease recognizing a NGG PAM site. A phylogenetic analysis of PBS1 dCTP deaminase and closely related T4 phage dCMP deaminase suggest possible evolutionary origins from bacterial dCDP deaminases. Overall, these findings suggest independent acquisition of dU‐DNA biosynthetic enzymes and pathways in the diverse phages, and support its protective function against different host‐encoded nucleases.

## Graphical abstract

Distinct enzymes including dCTP deaminases (Dcds), dTMP phosphatases (Dtms), and dTTP pyrophosphatases (Dtt) are found to be responsible for dU‐DNA biosynthesis in the phages PBS1, DSS3_VP1, and PhiR1‐37. Both PhiR1‐37‐derived and synthetic dU‐DNA are resistant to cleavage by restriction enzymes and Cas12a nuclease targeting dT‐containing sites. This study provides insights into biosynthetic pathways and the functional significance of phage dU‐DNA.

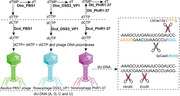

## 1 Introduction

Phages play a critical role in ecosystems, influencing the life cycles of bacteria and co‐evolving with their hosts through lysis, lysogeny and horizontal gene transfer.^[^ ^1^ ^]^ To defend against phages, bacteria have developed restriction‐modification (types I‐IV)^[^ ^2^ ^]^ and CRISPR‐Cas systems^[^ ^3^ ^]^ which function in recognizing and degrading foreign phage or plasmid DNA, while protecting their own genomes. Conversely, phages have evolved diverse strategies to evade restriction digestion, including the use of restriction‐site free DNAs,^[^ ^4^ ^]^ the use of DNA‐like proteins (Ocr^[^ ^5^ ^]^ and ArdA^[^ ^6^ ^]^) to inhibit restriction enzymes, and various types of DNA modification. DNA modification can be introduced post‐replicatively at the nucleic acid level, for example, methylation by phage‐encoded methyltransferases,^[^ ^7^ ^]^ or pre‐replicatively by synthesizing the modified nucleotides that are subsequently incorporated by DNA polymerase during replication.^[^ ^8^, ^9^ ^]^

The occurrence of dU‐DNA, in which thymidine (dT) is globally replaced with deoxyuridine (dU), was first identified in the genomes of the *Bacillus subtilis* (*B. subtilis*) phage PBS1 and its mutant PBS2.^[^ ^10^ ^]^ In uninfected *B. subtilis*, dUTP originating from ribonucleotide reduction is hydrolyzed by a specific dUTP pyrophosphatase (dUTPase) into dUMP, which is converted by thymidylate synthase to dTMP. The dUTPase activity keeps intracellular dUTP concentrations low,^[^ ^11^ ^]^ thereby minimizing mis‐incorporation of dUTP into DNA. Furthermore, bacterial uracil glycosylase catalyzes the removal of any mis‐incorporated dU in single‐ and double‐stranded DNA.^[^ ^9^ ^]^ By contrast, phage infection is accompanied by the induction of several proteins that disrupt host deoxynucleotide biosynthesis and regulation, promoting the synthesis of phage dU‐DNA instead of conventional dT‐DNA.

**Figure 1:**
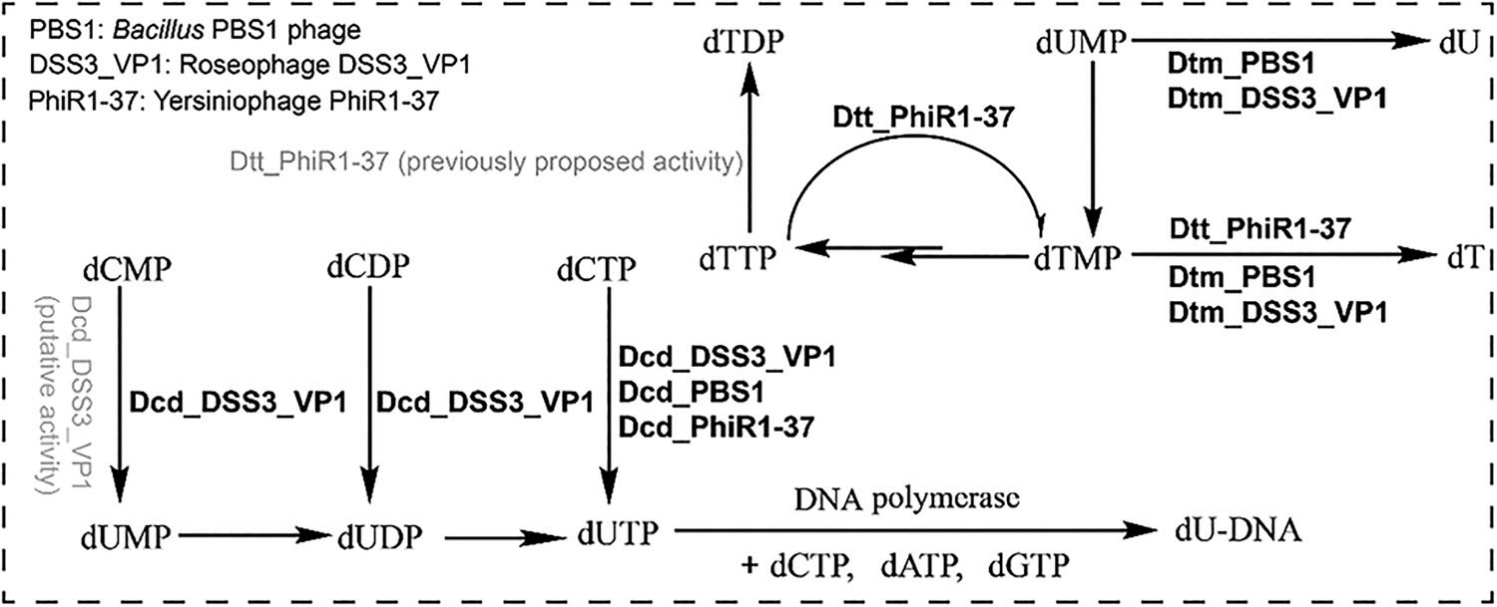
The biosynthetic pathway of bacteriophages deoxyuridine‐substituted DNA. The *Bacillus* phage PBS1 and Yersiniophage PhiR1‐39 dU‐DNA biosynthetic enzymes characterized here are highlighted in boldface. The previously proposed activities of Roseophage DSS3_VP1 putative dCMP deaminase (Uniprot: A0A7S5FQC0) and dTTP phosphatase (A0A7S5FQB6) are labeled in gray, while their revised experimentally determined activities are highlighted in boldface. The genes and enzymes identified and characterized in this study are Dcd (dCTP deaminase), Dtm (dTMP phosphatase), and Dtt (dTTP pyrophosphatase).

During PBS1/2 infection, the induced dCTP deaminase (Dcd)^[^ ^12^, ^13^ ^]^ and dUMP kinase^[^ ^14^ ^]^ promote the synthesis of dUTP as a precursor for phage dU‐DNA, while dTMP phosphatase (Dtm) depletes dT from the nucleotide pool to minimize its incorporation into phage DNA^[^ ^15^ ^]^ (**Figure** **1**). An induced 83 kDa dUTPase inhibitor further facilitates the accumulation of dUTP for phage DNA synthesis.^[^ ^11^ ^]^ While the *B. subtilis* DNA polymerase preferentially incorporates dTTP over dUTP, the PBS2‐induced DNA displays relaxed substrate specificity, incorporating dUTP and dTTP at comparable rates, likely an adaptation for dU‐DNA synthesis^[^ ^16^ ^]^ (Figure 1). Furthermore, dU‐DNA degradation is inhibited by the induction of an 18 kDa heat‐stable uracil glycosylase inhibitor.^[^ ^14^, ^17^ ^]^

dU‐DNA has also been identified in other diverse phages, including the Gram‐negative Yersiniophage PhiR1‐37,^[^ ^18^ ^]^ and Roseophages DSS3_VP1 and DSS3_PM1.^[^ ^19^ ^]^ In DSS3_VP1, dU‐DNA biosynthesis was proposed to involve a putative dCMP deaminase (Uniprot: A0A7S5FQC0) and dTTP phosphatase (A0A7S5FQB6)^[^ ^19^ ^]^ (Figure 1), a member of the HD hydrolase family^[^ ^20^ ^]^ that includes nucleotide phosphohydrolases, pyrophosphohydrolases, and triphosphohydrolases.^[^ ^9^ ^]^ The biosynthetic pathway for the Yersiniophage PhiR1‐37 dU‐DNA remains uncharacterized.

Although key enzymatic activities required for phage dU‐DNA biosynthesis have been detected, the corresponding genes and enzymes in diverse phages have yet to be identified and characterized. In this study, we present bioinformatic and biochemical analyses of enzymes involved in dU‐DNA biosynthesis in phages PBS1, DSS3_VP1, and PhiR1‐37, including distinct dC deaminases that generate the dU nucleotide precursors, and dT nucleotide phosphohydrolases that degrade dT nucleotides that compete for incorporation by DNA polymerase (Figure 1, **Table** **1**). We further assessed the resistance of Yersiniophage PhiR1‐37‐derived and synthetic dU‐DNA to cleavage by restriction enzymes and a CRISPR nuclease targeting dT‐containing sites, providing insights into the biosynthetic pathways and functional significance of phage dU‐DNA modification.

**Table 1:** The accession numbers and main reaction catalyzed by phage enzymes involved in dU‐DNA biosynthesis in this study.

Enzyme names	Accession numbers	Main reaction catalyzed
Dcd_PBS1	A0A223LD31	deamination of dCTP to dUTP
Dcd_ DSS3_VP1	A0A7S5FQC0	deamination of dCTP to dUTP
Dcd_PhiR1‐37	G4KK42	deamination of dCTP to dUTP
Dtm_PBS1	A0A223LEB8	hydrolyzes dTMP to dT
Dtm_ DSS3_VP1	A0A7S5FQB6	hydrolyzes dTMP to dT
Dtt_ PhiR1‐37	G4KK39	hydrolyzes dTTP to dTMP

Dcd: dCTP deaminase; Dtm: dTMP phosphatase; Dtt: dTTP pyrophosphatase.

## 2 Results

### 2.1 Identification and Characterization of a Dcd and Dtm in Bacillus Phage PBS1

The activities of Dcd and Dtm were previously detected during *Bacillus* phage PBS1 infection, although the corresponding genes remain unidentified. The genome of *Bacillus* phage PBS1 encodes an enzyme annotated as dCMP/CMP deaminase (A0A223LD31), exhibiting 38.15% sequence identity with T4 phage dCMP deaminase (DCTD, Uniprot: P16006, PDB 1VQ2),^[^ ^21^ ^]^ which we propose as a candidate Dcd. Sequence alignments and comparisons of its AlphaFold model to DCTD suggests that Dcd_PBS1 contains two conserved metal ion‐binding sites (Figure S1A,B, Supporting Information), with the Lewis acid catalytic metal ion (M1) coordinated by His102, Glu104, Cys130, and Cys133; and the structural metal ion (M2) coordinated by Cys22, Cys52, His92, Ser96, and Glu100.^[^ ^21^ ^]^ In addition, Dcd_PBS1 contains two conserved substrate‐interacting residues (Val27 and Asn43) that bind and stabilize the dC nucleoside moiety (Figure S1A,B, Supporting Information), consistent with a conserved catalytic mechanism. However, unlike DCTD, Dcd_PBS1 lacks a C‐terminal 14 amino acid, resulting in a more open active site that may accommodate the bulky triphosphate of its proposed substrate dCTP (Figure S1A,B, Supporting Information).

**Figure 2:**
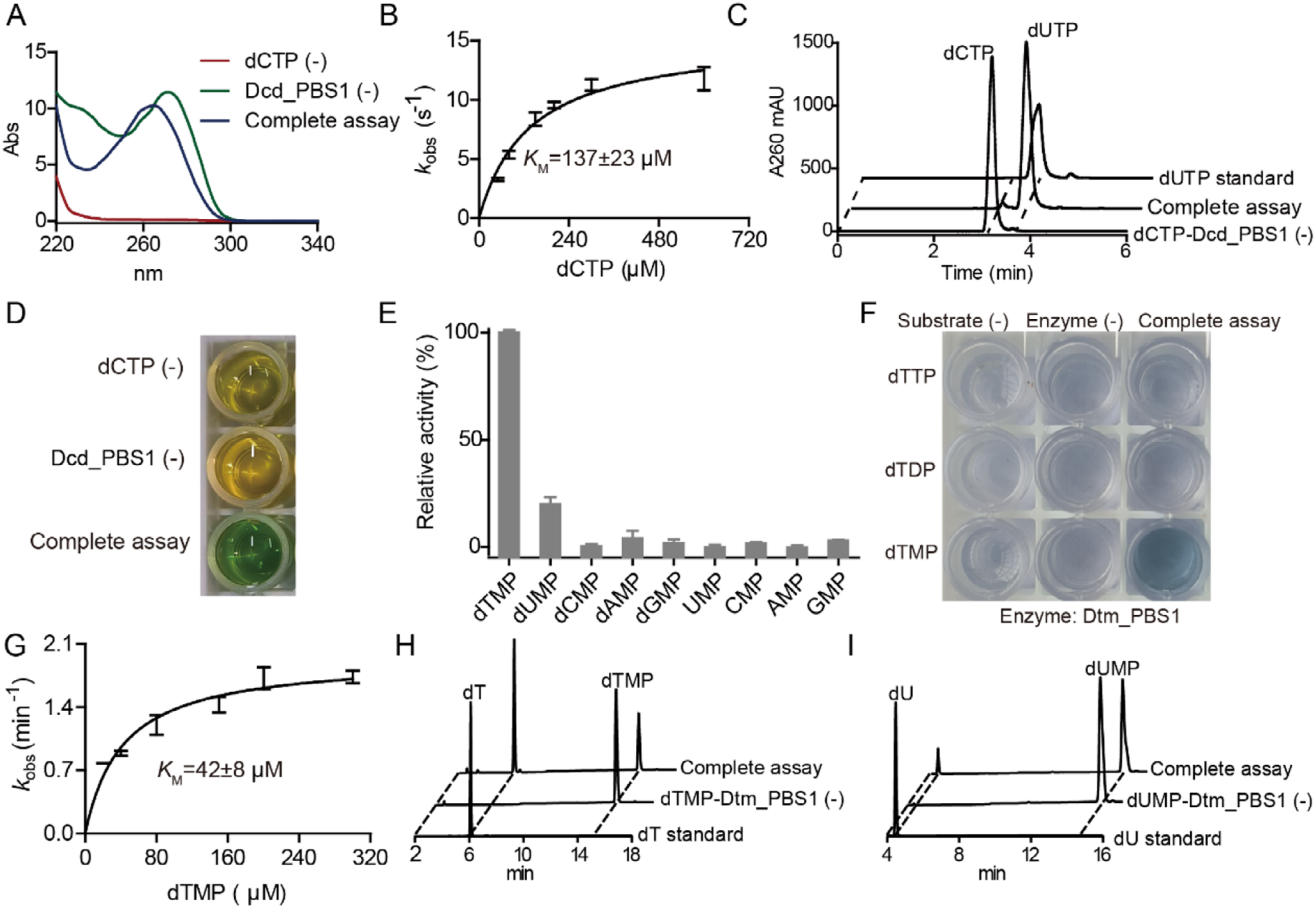
Dcd_PBS1 and Dtm_PBS1 activity assays. A) UV spectra of the Dcd_PBS1 assay with its substrate dCTP. B) Michaelis–Menten kinetics of Dcd_PBS1, varying the concentration of dCTP (*n* = 3). C) LC‐UV elution profile of Dcd_PBS1 assay, showing conversion of dCTP to dUTP. D) Images of a microtiter plate of salicylic acid‐hypochlorite colorimetric assays for Dcd_PBS1, showing ammonia release. E) Substrate specificity assay for Dtm_PBS1 with (d)NMPs (*n* = 3). F) Images of a microtiter plate of phosphomolybdate colorimetric assays for Dtm_PBS1 with dTTP, dTDP, or dTMP. G) Michaelis–Menten kinetics of Dtm_PBS1, varying the concentrations of dTMP (*n* = 3). H,I) LC‐UV elution profile of Dtm_PBS1 assay with dTMP or dUMP as substrate respectively. “(‐)” indicates negative controls omitting substrate or enzymes. All enzymatic assays were carried out in triplicate (*n* = 3). Data are presented as mean ± standard deviation (SD), with error bars indicating the SD.

The candidate Dcd_PBS1 was recombinantly produced and purified to homogeneity (Figure S2A, Supporting Information). Activity assays were conducted with Dcd_PBS1 as isolated or supplemented with various divalent metal ions.^[^ ^22^ ^]^ Incubation of Dcd_PBS1 with dCTP and Mn^2+^ resulted in time‐dependent changes in the absorption spectra, indicating a reaction occurring at the nucleobase (**Figure** **2**A). UV difference spectra, obtained by subtracting the starting material spectrum from that of the product, exhibited a time‐dependent decrease in absorbance at 230 and 281 nm, an increase at 258 nm (Figure S3A, Supporting Information), consistent with the conversion of dCTP into dUTP.^[^ ^22^ ^]^ Dcd_PBS1 was specific for dCTP, and inactive toward dCDP, dCMP, and CTP (Figure S3B, Supporting Information). Without the addition of divalent metal, enzyme activity was barely detectable, suggesting that the recombinant protein is purified mostly in its apo form. Highest activity was obtained with Mn^2+^ as the divalent metal cofactor, and the enzyme retained ≈90% activity with Mg^2+^ or Ni^2+^ (Figure S3C, Supporting Information), though we note in general that our experiments may not reflect the physiological metal ion, and do not rule out the involvement of endogenous bound metal ions. A spectrophotometric assay monitoring the consumption of dCTP^[^ ^22^ ^]^ was used to determine the Michaelis–Menten kinetic parameters for dCTP deamination (*k_cat_* = 15.4 ± 1 s^−1^, *K_M_* = 137 ± 23 µm) (Figure 2B). The Dcd_PBS1 reaction was further analyzed by HPLC and co‐elution with commercial standards, showing the consumption of dCTP and production of dUTP (Figure 2C). Release of NH_4_^+^ was detected using a salicylic acid‐hypochlorite colorimetric assay^[^ ^23^ ^]^ (Figure 2D; Figure S3D, Supporting Information).

To search for a candidate Dtm, we examined the genomes of *Bacillus* phages encoding Dcd and identified a conserved uncharacterized protein (A0A223LEB8). Despite low sequence identity (16%), AlphaFold modeling (Figure S4A,B, Supporting Information) revealed structural similarity to human mitochondrial deoxyribonucleotidase (PDB 1Z4K, nucleotidase family)^[^ ^24^ ^]^ which catalyzes the dephosphorylation of dTMP and dUMP.^[^ ^25^ ^]^ This candidate Dtm was recombinantly expressed and purified to homogeneity with an N‐terminal Trigger factor (TF) solubility tag (Figure S2A, Supporting Information). Biochemical assays revealed that A0A223LEB8 was indeed a dTMP phosphatase (Dtm_PBS1), with the highest activity obtained using Mg^2+^ as the divalent metal cofactor (Figure 2E–H; Figure S3E, Supporting Information, apparent *k_cat_* of 1.9 ± 0.1 min^−1^ and apparent *K_M_* of 42 ± 8 µm). Of the nucleotides tested, Dtm_PBS1 exhibited the highest activity with dTMP, with a lower for dUMP (Figure 2I), and limited activities for other (d)NMPs (Figure 2E). It was also inactive toward dTTP and dTDP (Figure 2F; Figure S3F, Supporting Information), with limited activity toward dUTP (Figure S3G, Supporting Information).

### 2.2 Identification and Characterization of a Dcd and Dtm in Roseophage DSS3_VP1

**Figure 3:**
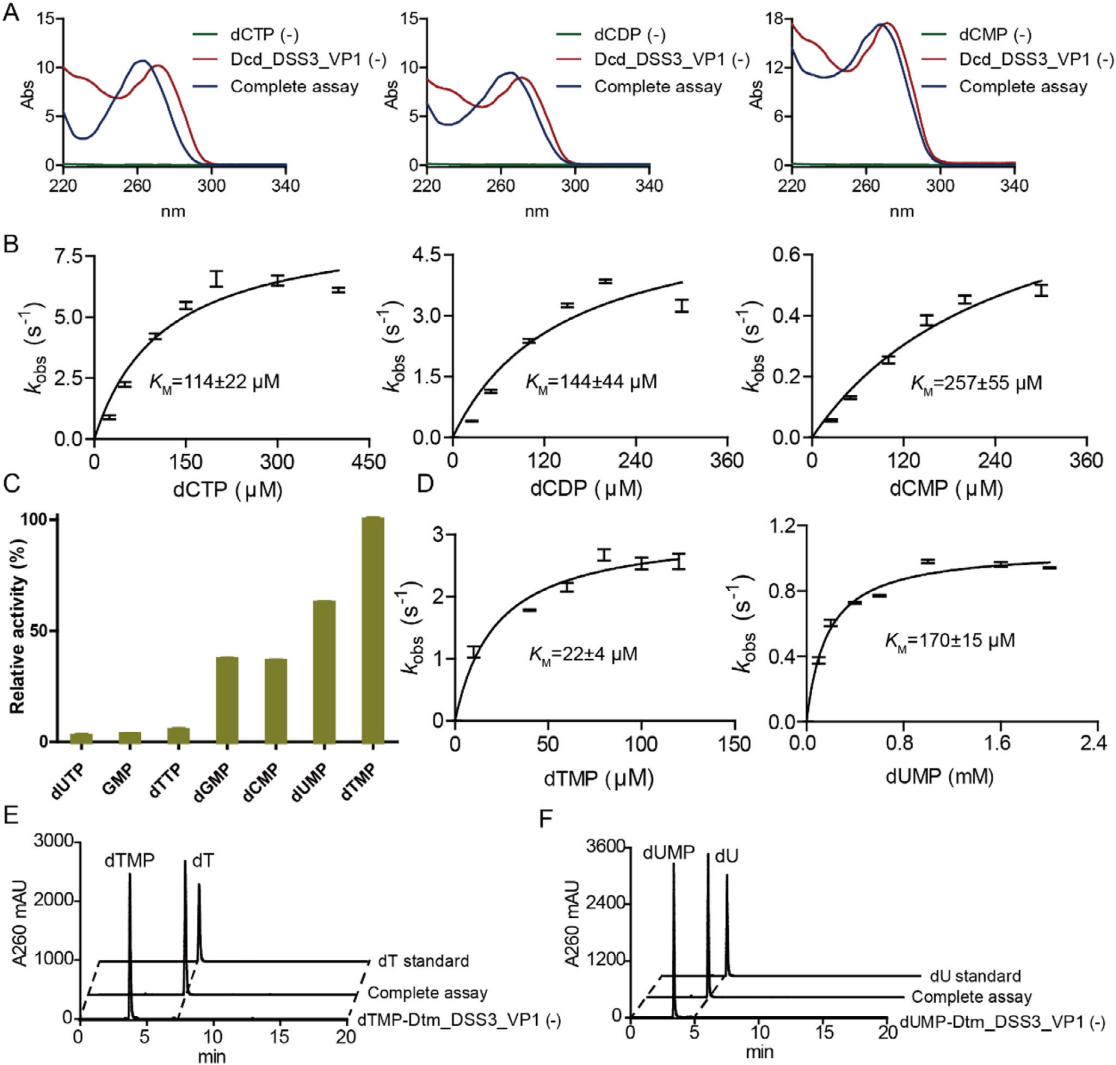
Dcd__DSS3_VP1 and Dtm_DSS3_VP1 activity assays. A) Substrate specificities of Dcd__DSS3_VP1 with dCTP, dCDP, or dCMP as substrate. B) Michaelis–Menten kinetics of Dcd__DSS3_VP1 varying the concentrations of dCTP, dCDP or dCMP respectively (*n* = 3). C) Substrate specificities of Dtm_PBS1 (*n* = 3). D) Michaelis–Menten kinetics of Dtm_PBS1 varying the concentrations of dTMP or dUMP respectively (*n* = 3). E,F) LC‐UV elution profile of Dtm_PBS1 assay with dTMP or dUMP as the substrate respectively. “(‐)” indicates negative controls omitting substrate or enzymes. All enzymatic assays were carried out in triplicate (*n* = 3). Data are presented as mean ± standard deviation (SD), with error bars indicating the SD.

The protein A0A7S5FQC0 was previously proposed as a candidate Roseophage dCMP deaminase.^[^ ^19^ ^]^ It exhibits limited homology to Dcd_PBS1 (A0A223LD31, 31% sequence identity), but is instead more closely related to human dCMP deaminase (UniProt: P32321, PDB:2W4L, 33% sequence identity),^[^ ^26^ ^]^ containing only the catalytic metal ion M1 and lacking the structural metal ion M2 site. Like Dcd_PBS1, the active‐site pocket of A0A7S5FQC0 is larger than that of phage T4 DCTD (Figure S1A, Supporting Information), suggesting that it might accommodate dCTP instead of dCMP. To investigate the substrate range of A0A7S5FQC0, the enzyme was recombinantly expressed with an N‐terminal MBP (Maltose‐binding protein) solubility tag, and purified to homogeneity (Figure S2B, Supporting Information). Incubation with either dCTP, dCDP, or dCMP resulted in time‐dependent changes in the absorption spectra, suggesting that this enzyme catalyzing the deamination of all three substrates (**Figure** **3**A). No activity was observed toward CTP (Figure S5A, Supporting Information). The highest activity was obtained with Mn^2+^ as the divalent metal cofactor, and the enzyme retained ≈80% activity when Co^2+^ or Mg^2+^ was used as the cofactor (Figure S5B, Supporting Information). A spectrophotometric assay monitoring the consumption of dCTP, dCDP or dCMP^[^ ^22^ ^]^ was used to determine the Michaelis–Menten kinetic parameters for the deamination reactions (Figure 3B), showing the highest activity with dCTP.

The protein A0A7S5FQB6, belonging to the HD hydrolase family, was previously identified as a candidate Roseophage dTTP phosphatase.^[^ ^19^ ^]^ To investigate the activity of this HD hydrolase, the enzyme was recombinantly expressed with an N‐terminal MBP solubility tag, and purified to homogeneity (Figure S2B, Supporting Information). Biochemical assays revealed that the enzyme is in fact a dTMP phosphatase (Dtm), with the highest activity obtained using Co^2+^ as the divalent metal cofactor (Figure 3C–E; Figure S5C, Supporting Information; apparent *k_cat_* of 3.1 ± 0.1 s^−1^ and apparent *K*_m_ of 22 ± 4 µm). Of the nucleotides tested, the enzyme had the highest activity for dTMP, with a lower activity for dUMP (Figure 3D,F; apparent *k_cat_* of 1 ± 0.1 s^−1^ and apparent *K*_m_ of 170 ± 15 µm), limited activity for other dNMPs and dTTP (Figure 3C), and no activity toward dUTP (Figure S5D–F, Supporting Information). Thus the assays indicate that this HD hydrolase acts as a Dtm, displaying functional similarity to Dtm_PBS1 despite originating from distinct enzyme families.

### 2.3 Identification and Characterization of a Dcd and Dtt from Yersiniophage PhiR1‐37

**Figure 4:**
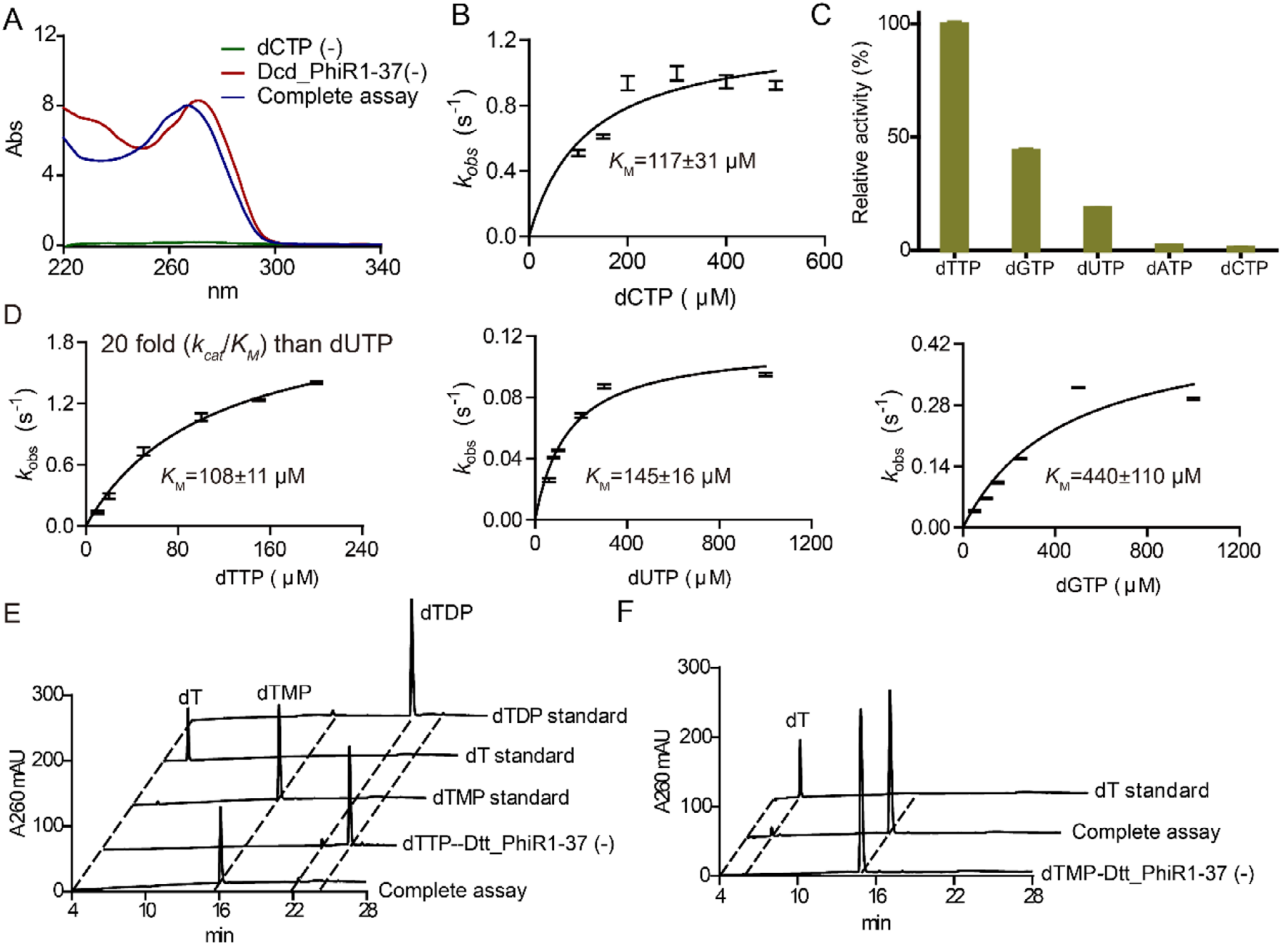
Dcd__PhiR1‐37 and Dtt_PhiR1‐37 activity assays. A) UV spectra of Dcd__PhiR1‐37 assays with dCTP as substrate. B) Michaelis–Menten kinetics of Dcd__PhiR1‐37 varying the concentrations of dCTP (*n* = 3). C) The substrate specificity assay for Dtt_PhiR1‐37 (*n* = 3). D) Michaelis–Menten kinetics of Dtt_PhiR1‐37 varying the concentrations of dTTP, dUTP, or dGTP respectively (*n* = 3). E,F) LC‐UV elution profile of Dtt_PhiR1‐37 assay with dTTP or dTMP as substrate respectively. “(‐)” indicates negative controls omitting substrate or enzymes. All enzymatic assays were carried out in triplicate (*n* = 3). Data are presented as mean ± standard deviation (SD), with error bars indicating the SD.

The genome of Yersiniophage PhiR1‐37 encodes an enzyme annotated as dCMP/CMP deaminase (G4KK42), limited homology to both Dcd_PBS1 (31%) and Dcd_DSS3_VP1 (27%), containing only the catalytic metal ion M1 and lacking the structural metal ion M2 site. To investigate the substrate selectively of Dcd__PhiR1‐37, the enzyme was recombinantly expressed with an N‐terminal MBP solubility tag, and purified to homogeneity (Figure S2C, Supporting Information). Substrate specificity assays demonstrated that Dcd__PhiR1‐37 is specific for dCTP and is inactive toward dCDP, dCMP and CTP (**Figure** **4**A; Figure S6A, Supporting Information). Without the addition of divalent metal, enzyme activity was barely detectable suggesting that the recombinant protein is purified mostly in its apo form. The highest activity was obtained with Mg^2+^ as the divalent metal cofactor, and the enzyme retained ≈80% activity with Mn^2+^ (Figure S6B, Supporting Information). A spectrophotometric assay monitoring the consumption of dCTP was used to determine the Michaelis–Menten kinetic parameters for dCTP deamination (*k_cat_* = 1.2 ± 0.1 s^−1^, *K_M_* = 117 ± 31 µm) (Figure 4B).

The genome neighborhood of Dcd__PhiR1‐37 contains an enzyme annotated as non‐canonical purine NTP pyrophosphatase (G4KK39), which we propose as a candidate dTTP pyrophosphatase (Dtt). Assays of the recombinant enzyme (Figure S2C, Supporting Information) showed that it is indeed a dTTP pyrophosphatase (Dtt_PhiR1‐37), with the highest activity obtained using Mg^2+^ as the divalent metal cofactor (Figure 4C–E; Figure S6C,D, Supporting Information, apparent *k_cat_* of 2.2 ± 0.1 s^−1^ and apparent *K_m_* of 108 ± 11 µm for dTTP). Dtt_PhiR1‐37 displayed lower pyrophosphatase activity with dGTP/dUTP (Figure 4C,D; Figure S5C,E,F, Supporting Information, apparent *k_cat_* of 0.4 ± 0.1 s^−1^ and apparent *K*_m_ of 440 ± 110 µm for dGTP, *k_cat_* of 0.1 ± 0 s^−1^ and apparent *K*_m_ of 145 ± 16 µm for dUTP), and little or no activities for dATP or dCTP (Figure 4C; Figure S6C, Supporting Information). Notably, the enzyme also displayed weak dTMP phosphatase activity (Figure 4F), which may complement its dTTP pyrophosphatase activity in removing dT from the nucleotide pool.

### 2.4 Characterization of dU‐DNA in Yersiniophage PhiR1‐37

**Figure 5:**
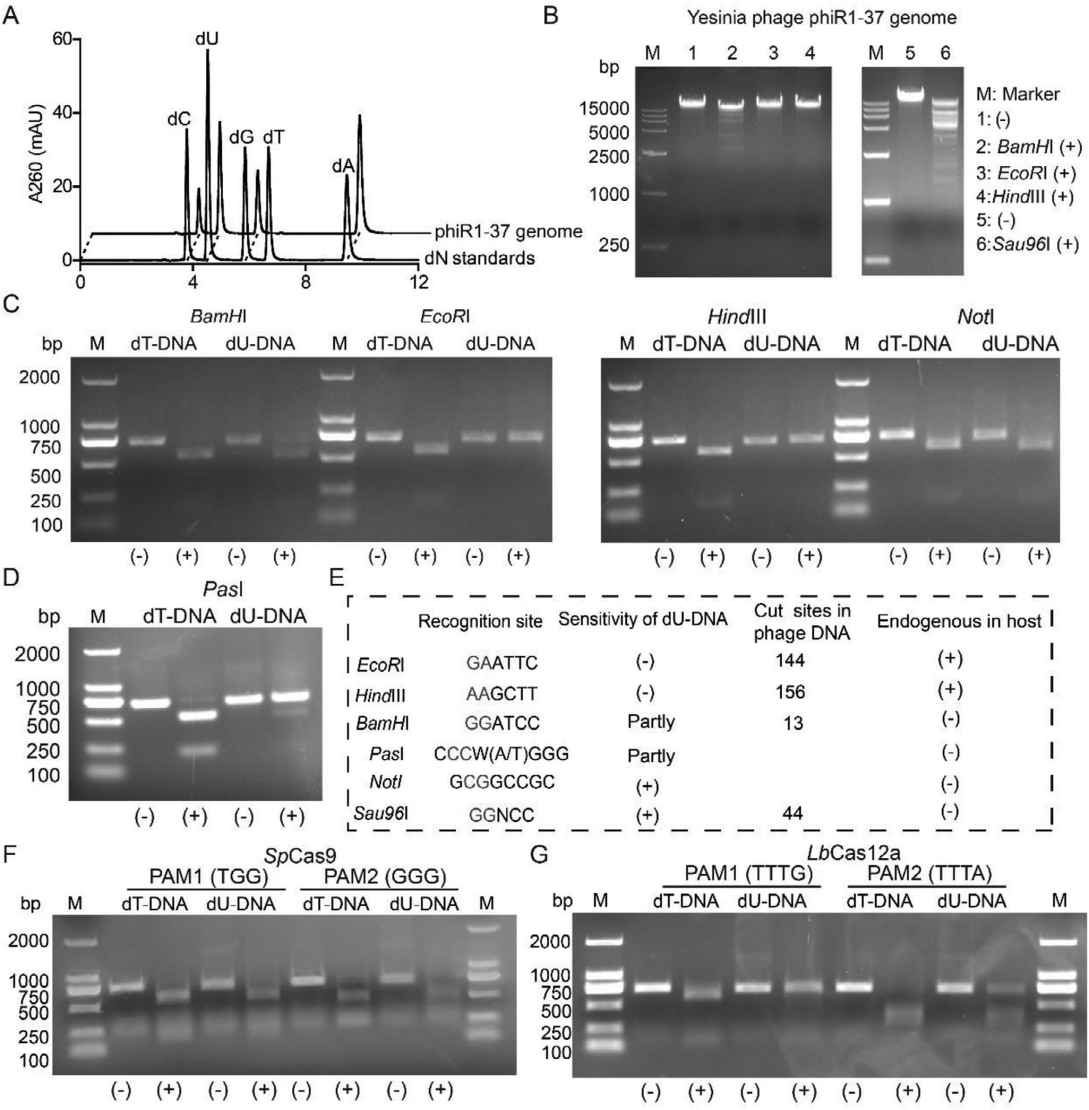
Validation of dU incorporation in the genome of Yesinia phage phi1‐37. A) LC‐UV detection of dU from the phage DNA hydrolysis products. B) Restriction enzyme digestions of the phage genomic DNA. ‐, control lane. C,D) Restriction enzymes were used to digest the PCR amplified dT‐DNA (normal DNA) and dU‐DNA fragment. ‐, control lane. E) The restriction enzyme table used in this study, the green label indicated the location of the cleavage site. F,G) *Sp*Cas9 and *Lb*Cas12a were used to digest the dT‐DNA (normal DNA) and dU‐DNA fragment respectively. ‐, control lane.

Identification of dUTP biosynthetic (Dcd) and dTTP‐degrading (Dtt/Dtm) enzymes in the three dU‐DNA phages points to a common strategy for achieving complete dU‐DNA substitution, raising the question of whether this modification serves a common function across these phages. The dU‐DNA of PBS 1‐related phages has been reported to be resistant to most restriction enzymes.^[^ ^27^ ^]^ To investigate whether this property extends to other dU‐DNA phages, we obtained and cultured the Yersiniophage PhiR1‐37, previously reported to contain dU‐DNA.^[^ ^18^ ^]^ The phage DNA was purified and digested with Nucleoside Digestion Mix,^[^ ^28^ ^]^ followed by liquid chromatography (LC)–UV spectrometric analyses. Using extinction coefficients to quantify the UV peaks yields the molar ratios of deoxynucleosides that fit Chargaff's rule, with the dU‐to‐dA ratio at 1.01 and the dC‐to‐dG ratio at 0.95, respectively (**Figure** **5**A), consistent with previous reports.^[^ ^18^ ^]^

Restriction endonuclease assays were conducted using purified Yersiniophage PhiR1‐37 DNA and synthetic dU‐DNA synthesized via PCR (using Taq polymerase and a dNTP mixture with dTTP replaced by dUTP). Both PhiR1‐37 genomic DNA and PCR‐amplified dU‐DNA were partially resistant to restriction enzymes with recognition sites containing one thymidine, and completely resistant to restriction enzymes with recognition sites containing two thymidines, including *EcoR*I and *Hind*III, which have close homologs present in its host *Yersinia enterocolitica* (*Y. enterocolitica*) O: 3.^[^ ^29^ ^]^ In contrast, the dU‐DNA samples were sensitive to *Not*I and *Sau96*I, which have GC‐only recognition sites (Figure 5B–E). Additionally, the PCR amplified dU‐DNA was also sensitive to *Sp*Cas9 endonuclease with TGG or GGG PAM sites (Figure 5F), but was partially resistant to digestion by *Lb*Cas12a endonuclease containing TTTG or TTTA PAM sites (Figure 5G ). Overall, these results support the proposal that dU‐DNA enables phages to evade restriction enzymes targeting thymidine‐containing, and CRISPR Cas systems that recognize thymidine‐containing PAM sequences.

### 2.5 Phylogenetic Analysis of Dcd_PBS1 Homologs and Characterization of Two Bacterial dCDP Deaminases

**Figure 6:**
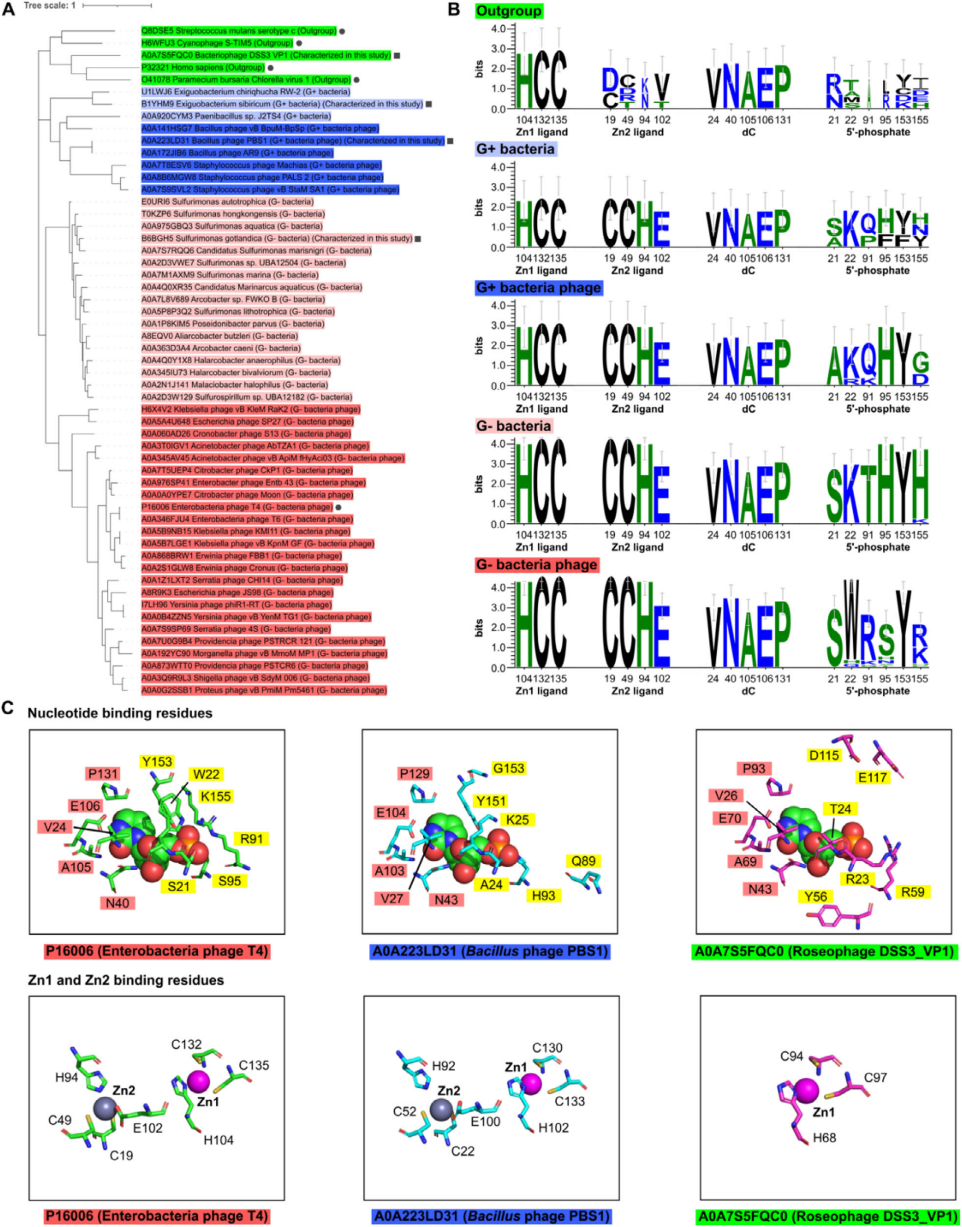
Phylogenetic analysis of Dcd and comparison of active site residues. Dcd homologs are color‐coded by taxonomic origin: light green (outgroup), light blue (Gram‐positive bacteria), dark blue (Gram‐positive bacteria phages), light red (Gram‐negative bacteria), dark red (Gram‐negative bacteria phages). A) Maximum likelihood phylogenetic tree of 50 Dcd homologs and structurally characterized members of the dCMP deaminase family (IPR016473, black circles). Entries characterized in this study are marked by black squares. B) Sequence logos illustrating conservation of metal‐ and substrate‐binding active site residues among Gram‐positive bacteria and their phages, along with Gram‐negative bacteria and their phages. Residue numbering corresponds to phage T4 dCMP deaminase. C) Active site structural models based on the crystal structure of phage T4 dCMP deaminase (PDB 1VQ2) and AlphaFold structures of Dcd_PBS1 and Dcd__DSS3_VP1. Substrate and metal ion positions were determined by structural superimposition with PDB 1VQ2. The highlighted labels show conservation of dC‐binding residues (red) and lack of conservation in alpha‐phosphate‐binding residues (yellow).

Although the Dcd_PBS1 from *Bacillus* phage PBS1 and dCMP deaminase from T4 phage differ in substrate specificity and biological function, their relatively high sequence identity (38.15%) prompted us to further explore the evolutionary origins of these enzymes. The MUSCLE software was used to construct a multiple sequence alignment that included the 50 homologs of Dcd identified via a BLAST search of the UniProt database (which includes Enterobacteria phage T4 dCMP deaminase), along with 4 other crystallographically characterized members of the dCMP deaminase family (IPR016473). The MEGA software was then used to construct a maximum likelihood phylogenetic tree, which was rooted at the mid‐point and plotted using the web‐based iTOL software. The tree contained two main branches, one comprising Gram‐positive Firmicutes bacteria (light blue, e.g., *Exiguobacterium sibiricum* (*E. sibiricum*), UniProt B1YHM9) and their phages (blue, including *Bacillus* phage Dcd_PBS1), the second comprising Gram‐negative Proteobacteria (light red, e.g., *Sulfurimonas gotlandica* (*S. gotlandica*), UniProt B6BGH5) and their phages (red, including the structurally characterized Enterobacteria phage T4 dCMP deaminase, Uniprot: P16006).^[^ ^21^ ^]^ The outgroup comprised the 4 remaining structurally characterized enzymes from various organisms, including dCMP deaminase from human (P32321), *Streptococcus mutans* (Q8DSE5), and cyanophage S‐TIM5 (H6WFU3), and dCMP/dCDP/dCTP deaminase from PBCV‐1 chlorovirus (O41078)^[^ ^30^ ^]^ (**Figure** **6**A).

The closest homologs of Dcd_PBS1 are present in‐ jumbo phages related to phage PBS1, including *B. subtilis* phage AR9, *Bacillus pumilus* phage vB_BpuM_BpSp, *Staphylococcus aureus* phages Machias, PALS_2, and vB_StaM_SA1 (Figure 6A). Some of these phages have also been reported to contain dU‐DNA,^[^ ^31^ ^]^ suggesting a similar role for Dcd in these phages in providing dUTP for phage DNA synthesis.

Analysis of the 50 close homologs of Dcd_PBS1 in the two main branches showed that the phage T4 dCMP deaminase Zn1 ligands (His104, Cys132, Cys135) and Zn2 ligands (Cys19, Cys49, His94, Glu102) are conserved (Figure 6B,C, Data S1, Supporting Information). In addition, residues interacting with the dC moiety of the dCMP substrate (Val24, Asn40, Ala105, Glu106, and Pro131) are also largely conserved. However, residues interacting with the 5′‐phosphate moiety differ significantly between phage T4 dCMP deaminase and Dcd_PBS1 (Figure 6B). Both the Gram positive and Gram‐negative bacterial sequences more closely resemble Dcd_PBS1, suggesting that they might also be dCTP deaminases.

To investigate the activity of the bacterial deaminases, we recombinantly expressed the enzymes from the Gram‐positive bacterium *E. sibiricum* (B1YHM9) and the Gram‐negative bacterium *S. gotlandica* (B6BGH5) with N‐terminal MBP solubility tags, and purified them to homogeneity (Figure S2D, Supporting Information). The *E. sibiricum* deaminase was active toward dCDP, dCTP, and dCMP, with the highest activity observed with dCDP, and with Co^2+^ as the metallocofactor (Figure S7A,B, Supporting Information). A spectrophotometric assay monitoring the consumption of dCDP (dCTP or dCMP) was used to determine the Michaelis–Menten kinetic parameters for dCDP (dCTP or dCMP) deamination (*k_cat_* = 21.2 ± 1.9 s^−1^, *K_M_* = 179 ± 31 µm for dCDP, *k_cat_* = 0.9 ± 0.1 s^−1^, *K_M_* = 186 ± 35 µm for dCTP, and *k_cat_* = 0.05 ± 0.01 s^−1^, *K_M _* = 415 ± 143 µm for dCMP) (Figure S7C, Supporting Information). The *S. gotlandica* deaminase was active toward dCDP, dCMP, and dCTP, with the highest activity observed with dCDP, and with Mn^2+^ as the metallocofactor (Figure S7D,E, Supporting Information). The Michaelis–Menten kinetic parameters for dCDP (dCMP or dCTP) deamination were likewise measured (*k_cat_* = 0.5 ± 0.1 s^−1^, *K_M _* = 271 ± 90 µm for dCDP, *k_cat_* = 0.28 ± 0.05 s^−1^, *K_M _* = 661 ± 178 µm for dCMP, and *k_cat_* = 0.06 ± 0.01 s^−1^, *K_M _* = 293 ± 88 µm for dCTP) (Figure S7F, Supporting Information). Overall, the biochemical result and phylogenetic analysis suggest that the highly specific phage T4 dCMP deaminase and Dcd_PBS1 may have evolved from promiscuous bacterial dCDP deaminases.

## 3 Discussion

Our research on *Bacillus* phage PBS1, Roseophage DSS3_VP1, and Yersiniophage PhiR1‐37, has identified and characterized dC nucleotide deaminases and dT nucleotide phosphohydrolases, playing key roles in the synthesis of their dU‐DNA genomes. All three deaminases belong to the metal‐dependent “dCMP deaminase” family (IPR016473), which includes human and T4 phage dCMP deaminase (Figure S8, Supporting Information), and are structurally related to human DNA cytosine deaminases AID and APOBEC. Of the deaminases, Dcd_PBS1 and Dcd__PhiR1‐37 are specific for dCTP, while Dcd__DSS3_VP1 is more promiscuous, displaying highest activity with dCTP and lower activity with dCDP and dCMP (**Table** **2**). These enzymes are distinct from the *E. coli* dCTP deaminase, which belongs to the metal‐independent “dCTP deaminase” family (IPR011962).^[^ ^22^ ^]^

**Table 2:** The kinetic parameters of phage enzymes involved in dU‐DNA biosynthesis.

Enzyme names	Substrate and cofactor	kinetic parameters
Dcd_PBS1	dCTP, Mn^2+^	*k_cat_ = *15.4 ± 1 s*^−1^, K_M _= *137 ± 23 µm
Dcd_ DSS3_VP1	dCTP, Mn^2+^	*k_cat_ = *8.9 ± 0.7 s*^−1^, K_M _= *114 ± 22 µm
Dcd_ DSS3_VP1	dCDP, Mn^2+^	*k_cat_ = *5.6 ± 0.8 s*^−1^, K_M _= *144 ± 44 µm
Dcd_ DSS3_VP1	dCMP, Mn^2+^	*k_cat_ = *1 ± 0.1 s*^−1^, K_M _= *257 ± 55 µm
Dcd_PhiR1‐37	dCTP, Mg^2+^	*k_cat_* = 1.2 ± 0.1 s^−1^, *K_M _* = 117 ± 31 µm
Dtm_PBS1	dTMP, Mg^2+^	*k_cat_* = 1.9 ± 0.1 min^−1^, *K_M_* = 42 ± 8 µm
Dtm_ DSS3_VP1	dTMP, Co^2+^	*k_cat_ = *3.1 ± 0.1 s*^−1^, K_M _= *22 ± 4 µm
Dtm_ DSS3_VP1	dUMP, Co^2+^	*k_cat_ = *1 ± 0.1 s*^−1^, K_M _= *170 ± 15 µm
Dtt_ PhiR1‐37	dTTP, Mg^2+^	*k_cat _* = 2.2 ± 0.1 s^−1^, *K_M_* = 108 ± 11 µm
Dtt_ PhiR1‐37	dGTP, Mg^2^	*k_cat_ = *0.4 ± 0.1 s^−1^, *K_M_ *= 440 ± 110 µm
Dtt_ PhiR1‐37	dUTP, Mg^2+^	*k_cat_ *= 0.1 ± 0 s^−1^, *K_M_ *= 145 ± 16 µm

Dcd: dCTP deaminase; Dtm: dTMP phosphatase; Dtt: dTTP pyrophosphatase.

Notably, the three phage deaminases studied share low pairwise sequence identity (≈29%), with Dcd_PBS1 showing higher identity (≈38%) to the dCMP deaminase of the hmdC‐DNA–containing T4 phage, than to the other two deaminases from dU‐DNA phages. Our phylogenetic and biochemical studies suggest that Dcd_PBS1 and T4 phage dCMP deaminase may have separately evolved from promiscuous bacterial dCMP/dCDP/dCTP deaminases. Homologs of Dcd_PBS1 are found in several other jumbo phages infecting Gram‐positive bacteria, some of which are known to contain dU‐DNA. Like the human and T4 phage dCMP deaminases,^[^ ^21^, ^32^ ^]^ *Bacillus* phage PBS1 Dcd is negatively regulated by dUTP (Figure S9, Supporting Information). While eukaryotic and bacterial dCMP deaminases generate dUMP for dTMP synthesis via thymidylate synthase, dCTP deaminases in dU‐DNA phages may represent an adaptation to directly produce dUTP for genome incorporation, while also maintaining the dCTP/dUTP ratio.

Unlike the deaminases, the three phage dT nucleotide phosphohydrolases are from different families. The phage PBS1 Dtm belongs to the deoxyribonucleotidase family (PF06941), phage DSS3_VP1 Dtm belongs to the HD hydrolase yfbR family (PF12917), while the phage PhiR1‐37 dTTP pyrophosphatase belongs to the nucleotide triphosphate pyrophosphatase HAM1 family (PF01725). The physiological source of dTMP is thymidylate synthase, and the activity the Dtms aligns with their proposed role in removing dT from the nucleotide pool. Both Dtms show minimal dUTP hydrolase activity, thus preserving the dUTP pool. The Dtm_PBS1 exhibits relatively low activity, but high specificity for dTMP. By contrast, the Dtm_ DSS3_VP1 displays higher activity, but low specificity, with significant dUMPase activity (50% that of dTMP), suggesting a possible tradeoff between activity and specificity in the evolution of Dtms (Table 2). In addition, the promiscuous dUMPase activity suggests that Roseophage DSS3_VP1 may require a mechanism for preventing the conversion of dU nucleotides into dUMP, possibly involving a dUTPase inhibitor similar to phage PBS2.

The most widely accepted function of phage DNA modifications, including dU‐DNA, is to evade bacterial immune defenses such as restriction enzymes. To be effective, this function would require near‐complete substitution of the phage genome. In dZ‐DNA phages, complete replacement of dA with dZ was found to require enzymes that both synthesize the dZTP precursor (PurZ and dGTP diphosphatase) and degrade competing dATP (dATP triphosphatase).^[^ ^9^ ^]^ In our study of dU‐DNA phages, the identification of enzymes for dUTP biosynthesis (Dcd) and dTTP degradation (Dtt/Dtm) supports an analogous strategy to promote complete dU substitution. Both Yersiniophage PhiR1‐37 genomic dU‐DNA and PCR‐synthesized dU‐DNA exhibited resistance against restriction enzymes with dT‐containing recognition sites, with the stronger resistance observed for enzymes with recognition sites containing multiple dT. Notably, dU‐DNA also exhibited partial resistance to cleavage by *Lb*Cas12a, which recognizes a TTTV PAM site. Structural studies of the *Lb*Cas12a ternary complex by Yamano et al. revealed that the 5‐methyl groups of dT(−3) and dT(−4) interact with specific protein residues of Cas12a, contributing to the specific recognition of the TTTA PAM duplex.^[^ ^33^ ^]^ By contrast, the PCR amplified dU‐DNA was sensitive to the *Sp*Cas9 endonuclease, which recognizes an NGG PAM site. In previous studies examining the effect of DNA base modifications on CRISPR/Cas cleavage, assays of *Lb*Cas12a and *Sp*Cas9 with 2,6‐diaminopurine‐modified DNA (dZ‐DNA) showed partial resistance to *Lb*Cas12a but no effect to *Sp*Cas9.^[^ ^34^ ^]^ The activity of *Sp*Cas9 was unaffected by DNA cytosine and adenine methylation^[^ ^35^ ^]^ but was inhibited by the 7‐deazapurine substituted DNA, which was attributed to the essential role of the purine N7‐atom in NGG PAM site recognition.^[^ ^36^ ^]^

In conclusion, our identification and biochemical characterization of phage dU‐DNA synthesis enzymes expands our understanding of how phages remodel nucleotide metabolism to evade host defenses. The distant relationship between dU‐DNA biosynthetic enzymes in these phages supports the notion of independent acquisition of dU‐DNA synthesis pathways. Further studies are needed to define the complete set of proteins involved and to explore the convergent evolution of these biochemical strategies. These findings not only clarify the molecular basis of dU‐DNA synthesis in diverse phages but also enable bioinformatic prediction of dU‐DNA phages in genomic and metagenomic data, providing valuable tools for phage synthetic biology and therapeutic applications.

## 4 Experimental Section

### General

Lysogeny broth (LB) was purchased from Sangon Biotech (Shanghai, China). Amylose Resin (E8021V‐10 mL) and 5‐methyl‐dCTP (N0356S), *BamH*I‐HF (R3136V), *Hind*III‐HF (R3104V), *EcoR*I‐HF (R3101V), *Not*I‐HF (R3189V), and EnGen Lba Cas12a (Cpf1) nuclease (M0653S) were ordered from New England Biolabs, Inc, (NEB, USA). *Pas*I (ER1861‐200 units) restriction endonuclease was purchased from Thermo Fisher. dCTP, dCDP, dCMP, CTP, dUTP, dUMP, dTTP, dTMP, dGMP, GMP, dT, and dU were purchased from Beyotime (Shanghai, China), Aladdin (Shanghai, China), and Macklin (Shanghai, China). The sgRNA1 (5′‐UAAUUUCUACUAAGUGUAGAUCCGCUUGCGGUGCCAUUAAU‐3′, PAM: TTTG) and sgRNA2 (5′‐UAAUUUCUACUAAGUGUAGAUUGGGCUUUGGCAAUUGCAUUCAU, PAM: TTTA‐3′) for *Lb*Cas12a, and the sgRNA3 (5′‐UGUGAUAAAUUCAUGAAGCGCGAGUUUUAGAGCUAGAAAUAGCAAGUUAAAAUAAGGCUAGUCCGUUAUCAACUUGAAAAAGUGGCACCGAGUCGGUGCUUUU‐3′, PAM: TGG) and sgRNA4 (5′‐AAAUAUCUUCGAUCCGUGGGGCUGUUUUAGAGCUAGAAAUAGCAAGUUAAAAUAAGGCUAGUCCGUUAUCAACUUGAAAAAGUGGCACCGAGUCGGUGCUUUU‐3, PAM: GGG) for *Sp*Cas9 were synthesized by Tsingke Biotechnology. UV/UV–vis spectroscopic measurements were made using a NANODROP ONE (Thermo SCIENTIFIC), or using a Biotek Synergy 2 reader for 96‐well plates. All enzyme activity assays were performed at room temperature, and enzyme activity results were analyzed by GraphPad Prism 5.0.

### Identification of Dcd in Phage PBS1

The genome DNA sequence of phage PBS1 (NC_04 3027) was download from NCBI, and use dCMP T4 deaminase (DCTD, Uniprot: P16006)^[^ ^21^ ^]^ as a reference sequence, a putative dCTP deaminase (Dcd_PBS1)was identified in phage PBS1 by homologous sequence alignment. AlphaFold 3^[^ ^37^ ^]^ online protein structure modeling tools were employed to generate a Dcd_PBS1 structure model. With the reported crystal structure of dCD (PDB ID: 1VQ2)(21) as a template, the key amino acid residues at the active site of Dcd_PBS1 were examined and compared, and the ChimeraX (https://www.cgl.ucsf.edu/chimerax/) software was used for protein structure visualization.

### Gene Syntheses, Cloning, Expression, and Purification of Dcds, Dtms, Dtt, and Bacterial Cytidine Nucleotide Deaminase

The codon‐optimized gene fragments of Dcd_PBS1 (Uniprot: A0A223LD31), Dtm_PBS1 (A0A223LEB8); Dcd__DSS3_VP1 (A0A7S5FQC0), Dtm_DSS3_VP1 (A0A7S5FQB6); Dcd__PhiR1‐37 (G4KK42), Dtt__PhiR1‐37 (G4KK39); *E. sibiricum* cytidine nucleotide deaminase (B1YHM9) and *S. gotlandica* cytidine nucleotide deaminase (B6BGH5) were synthesized by General Biol Inc. (Anhui, China), and inserted into HMT plasmid by Gibson assembly, for expression with an N‐terminal His_6_‐MBP tag.^[^ ^38^ ^]^ The proteins were expressed and purified using a similar process as previously described using an amylose affinity column.^[^ ^38^ ^]^

The Dtm (A0A223LEB8) from phage PBS1 was not successfully expressed and purified as the above method. Then, its gene sequence was amplified by PCR (F: 5′‐CCTGTACTTCCAGGGCATGTTCAGCATTAAGGAACCG‐3′, R: 5′‐GAATTCGGATCCGCGTTAAACGGCTTTTTCTTCGGTTT‐3′) and cloned into the pCold TF plasmid vector (Takara Code No. 3365) by Gibson assembly. The resulting plasmid pCold TF‐Dtm_PBS1 contains in tandem: a His_6_‐tag, TF, and a TEV protease cleavage site, followed by the construct of phage PBS1 Dtm, and was verified by sequencing. The. *E. coli* BL21 (DE3) cells were transformed with the pCold TF‐Dtm_PBS1 and plated on LB agar supplemented with 20 µg mL^−1^ ampicillin. The protein was expressed and purified using a similar process as previously described using a Ni‐NTA affinity column.^[^ ^39^ ^]^ The concentrations of purified proteins were calculated from their absorption at 280 nm using a NANODROP ONE (Thermo SCIENTIFIC) Purified proteins were analyzed on a 10% SDS polyacrylamide gradient gel, and Coomassie stains were used to visualize them.

### Enzyme Assays of Dcds

The Dcds catalyzed conversion of dCTP/dCDP/dCMP to dUTP/dUDP/dUMP was monitored by UV spectroscopy.^[^ ^22^ ^]^ The substrate specificity assay was performed with 40 µL reaction mixture containing 50 mm Tris‐HCl, pH 7.5, 1 mm dCTP (or substrate analogs including dCDP, dCMP and CTP), 0.2 µM of Dcd_PBS1 (Dcd__PhiR1‐37, Dcd__DSS3_VP1, *E. sibiricum* cytidine nucleotide deaminase; or 0.5 µm of *S. gotlandica* cytidine nucleotide deaminase) and 2 mm Mn^2+^ (Mg^2+^ for Dcd__PhiR1‐37 or Co^2+^ for *E. sibiricum* cytidine nucleotide deaminase) was incubated for 10 or 30 min, and the time‐dependent dCTP‐*Bp*dCTPase assays were incubated for 0–6 min, followed by UV absorbance scan from 220 to 350 nm.

For the metal cofactor specificity assay, a 200 µL reaction mixture, containing 50 mM Tris‐HCl pH 7.5, 0.25, or 0.5 mm dCTP, 2 mm Mn^2+^ (or other divalent metal ions ZnSO_4_.7H_2_O, CoCl_2_.6H_2_O, MgCl_2_.6H_2_O, Ni_2_SO_4_, or CaCl_2_.2H_2_O), and 0.2 µm of Dcd_PBS1 (Dcd__PhiR1‐37 or 0.1 µm of Dcd__DSS3_VP1; 0.2 µm of *E. sibiricum* cytidine nucleotide deaminase or 0.5 µm of *S. gotlandica* cytidine nucleotide deaminase) was incubated in a 96 UV transparent plate (Corning 3635), was monitored for 0–5 min at 284 nm.^[^ ^22^ ^]^

To determine the Michaelis–Menten parameters for dCTP/dCDP/dCMP deamination, the absorbance at 284 nm of a 200 µL reaction mixture, including 50 mm Tris–HCl, pH 7.5, 0.02 µm of Dcd_PBS1 (0.2 µm of Dcd__PhiR1‐37 or 0.05–0.8 µm Dcd__DSS3_VP1; 0.05–4 µm of *E. sibiricum* cytidine nucleotide deaminase or 0.5–4 µm of *S. gotlandica* cytidine nucleotide deaminase), 0–0.6 mm of dCTP (0–0.5 mm of dCDP or 0–0.5 mm of dCMP) and 2 mm Mn^2+^ in a 96 UV transparent plate (Corning 3635), was monitored for 2 min. ΔA284 nm and the extinction coefficient of 3800 M^−1^ cm^−1[^ ^22^ ^]^ were used to calculate the reaction rates. GraphPad Prism 5.0 was used to extract the kinetic parameters.

### Salicylic Acid‐Hypochlorite Spectrophotometry Analyses of the Dcd_PBS1 Reaction

NH_4_^+^/NH_3_ was detected by salicylic acid‐hypochlorite spectrophotometry using a previously described method.^[^ ^23^ ^]^ A 200 µL reaction mixture containing 0.5 mm dCTP, 2 mm Mn^2+^, and 1 µm Dcd_PBS1 was incubated for 10 min at RT. Negative controls omitting or dCTP or Dcd_PBS1 were also performed. An initial reaction sample of 60 µL was added to the 96‐well plate and followed by 100 µL of “salicylate mixture” and 80 µL of “hypochlorite solution”.^[^ ^23^ ^]^ Assays were incubated for 15 min at 37 °C and measured at 620 nm with a Biotek Synergy 2 reader.

### Enzyme Assays of Dtms and Dtt_PhiR1‐37

For the metal cofactor specificity assay of Dtms or Dtt_PhiR1‐37, a 100 µL reaction mixture, containing 50 mm Tris‐HCl pH 7.5, 0.5 mm dTMP, 2 mm Mg^2+^ (or other divalent metal ions ZnSO_4_.7H_2_O, MnCl_2_.4H_2_O, CoCl_2_.2H_2_O, Ni_2_SO_4_, CuSO_4_.5H_2_O, or CaCl_2_.2H_2_O), and 2 µm Dtm_PBS1 (0.5 µm Dtm_DSS3_VP1 or 2 µm Dtt_PhiR1‐37) was incubated for 30 min. The substrate specificity assay was performed with a 100 µL reaction mixture containing 50 mm Tris‐HCl, pH 7.5, 0.5 mm dTMP (or substrate analogs including dUMP, dCMP, dGMP, GMP, dTTP, and dUTP), 2 µm of Dtm_PBS1 (0.5 µm of Dtm_DSS3_VP1), and 2 mm Mg^2+^ (or Co^2+^ for Dtm_DSS3_VP1) was incubated for 15 min. To determine the Michaelis–Menten parameters for dTMP and dUMP phosphohydrolase reactions, a 100 µL reaction mixture, containing 50 mm Tris‐HCl, pH 7.5, 100–0 µm dTMP (or 2–0 mm dUMP), 2 mm Co^2+^, and 0.02 or 0.2 µm of Dtm_PBS1 or Dtm_DSS3_VP1 was incubated for 0–8 min at RT. The product of phosphate was quantified using a colorimetric phosphomolybdate assay.^[^ ^40^ ^]^

For the substrate specificity assay of Dtt_PhiR1‐37, a 100 µL reaction mixture containing 50 mm Tris‐HCl, pH 7.5, 0.5 mm dTTP (or other dNTPs), 2 µm of Dtm_PBS1, 0.01 U IPP1 (Beyotime‐R7025M), and 2 mm Mg^2+^ was incubated for 20 min. To determine the Michaelis–Menten parameters for dTTP, dGTP, and dUTP pyrophosphohydrolase reactions, a 100 µL reaction mixture, containing 50 mm Tris‐HCl, pH 7.5, 1000–0 µm dGTP (dUTP or 200–0 µm dTTP), 2 mm Mg^2+^, 0.1–1 µm Dtt_PhiR1‐37, and 0.001 U IPP1 was incubated for 0–12 min at RT. The product of phosphate was quantified using a colorimetric phosphomolybdate assay.

### High Performance Liquid Chromatography Analyses of the Dcd_PBS1 Reaction

A 300 µL reaction mixture containing 20 mm Tris‐HCl, pH 7.5, 1 mm dCTP, 2 mm Mn^2+^, and 0.2 µm Dcd_PBS1 was incubated for 2 h at RT. Negative controls omitting Dcd_PBS1 were also performed. After incubation, the reaction solution was subjected to centrifugation (15 000 × g for 5 min at 4 °C). The supernatant was analyzed using an Agilent 1260 Infinity (USA) HPLC. The LC separation was performed on a Syncronis AQ (150 mm × 4.6 mm, 3 µm) column with a flow rate of 0.5 mL min^−1^ at RT, with 20 mm NH_4_H_2_PO_4_ pH 5.35 as mobile phase^[^ ^41^ ^]^ and eluted for 10 min. The wavelength of the UV detection was 260 nm.

### High Performance Liquid Chromatography Analyses of the Dtm_DSS3_VP1 Reaction

A 300 µL reaction mixture containing 20 mm Tris‐HCl, pH 7.5, 2 mm dTMP (dUMP or dUTP), 2 mm Co^2+^, and 2 µm Dtm was incubated for 0.5 h at RT. Negative controls omitting Dtm were also performed. After incubation, the reaction solution was followed by centrifugation (15 000 × g for 5 min at 4 °C). The supernatant was analyzed using an Agilent 1260 Infinity (USA) HPLC. The LC separation was performed on a Poroshell 120 EC‐C18 (150 mm × 4.6 mm, 4 µm) column with a flow rate of 0.5 mL min^−1^ at RT. NH_4_AC (10 mm) pH 4.6 in water (solvent A) and methanol (solvent B) were employed as the mobile phase.^[^ ^9^ ^]^ A gradient of 0–20 min 20–40% B was used. The sample injection volume was 10 µL, and the UV detector was set at 260 nm.

### High Performance Liquid Chromatography Analyses of the Dtm_PBS1 and Dtt_PhiR1‐37 Reaction

For the Dtm_PBS1 assay, a 300 µL reaction mixture containing 20 mm Tris‐HCl, pH 7.5, 2 mm dTMP (dUMP, dTTP, or dUTP), 2 mm Mg^2+^, and 2 µm Dtm_PBS1 was incubated for 1 h at RT. Negative controls omitting Dtm_PBS1 were also performed. For the Dtt_PhiR1‐37 assay, a 300 µL reaction mixture containing 20 mm Tris‐HCl, pH 7.5, 0.5 mm dTTP (dTMP, dGTP, or dUTP), 2 mm Mg^2+^, and 0.5–2 µm Dtm_PBS1 was incubated for 1 h at RT. Negative controls omitting Dtt_PhiR1‐37 were also performed. The reaction solution then filtered prior to HPLC assay using an Agilent 1260 Infinity (USA) HPLC. The LC separation was performed on an Agilent Polaris 3 C18‐A column (4.6 × 150 mm, product number 200 113 523) column with a flow rate of 1 mL min^−1^ at RT. TBAH (10 mm Tetrabutylammonium hydroxide) and 10 mm KH_2_PO_4_ in water (solvent A) and 10 mm TBAH in methanol (solvent B) were employed as mobile phase. A gradient of 0–25 min 5–50% B, 25–27 min 50%B, 27–27.1 min 50–95% B, 27.1–30.1 min 95%B, 30.1–30.2 min 95–5%B, and 30.2–40.2 min 5%B was used. The sample injection volume was 10 µL, and the UV detector was set at 254 nm.

### Yersiniophage PhiR1‐37 Genomic DNA Extraction

Yersiniophage PhiR1‐37 (DSM 23 247) and *Y. enterocolitica* (DSM 23 247) were purchased from DSMZ (German Collection of Microorganisms and Cell Cultures GmbH). PhiR1‐37 (10^9^ PFU mL^−1^) to log phase (OD_600_ 0.6–0.8) of *Y. enterocolitica* at a volume ratio of 1 to 4 was grown for 7–8 h in DSMZ Medium 220 at a 28 °C incubator. Then, the cell debris was removed by using a centrifuge at 8000 × g for 10 min at 4 °C. Genomic DNA of the Yersiniophage PhiR1‐37 was extracted using a Lambda Phage Genomic DNA Kit (Zoman Biotech, ZP317‐1).

### HPLC Spectrometric Analyses of the Yersiniophage PhiR1‐37 Genomic DNA

The genomic DNA from Yersiniophage PhiR1‐37 was enzymatically digested by Nucleoside Digestion Mix (NEB M0649S) overnight at 37 °C and separated on Amicon Ultra‐0.5 mL 3 K centrifugal filters (Millipore). The flow‐through was analyzed using an Agilent 1260 Infinity II instrument (Agilent Technologies, CA, and USA) as previously described method for phage Z‐genome.^[^ ^9^ ^]^ The standard compounds include commercially available deoxynucleosides (dA, dT, dC, dG, and dU)

### PCR Amplification of dU‐DNA Fragment with 725 bp

The reaction system contained 20 ng HMT‐Dcd_PBS1, 0.2 mm dNTPs (dTTP) or 0.2 mm dNTPs (dUTP), 0.5 µm forward primer (ATGACCAATAAGTGGGATCTGATGT), 0.5 µm reverse primer (CAAAAAACCCCTCAAGACCCGTT), 1 × Taq reaction buffer, and 1 µL Taq DNA polymerase (NEB‐M0273S). Reactions were performed following the manufacturer's instructions on a T100 Thermal Cycler (BIO‐RAD). The PCR products were purified using a StarPrep Gel Extraction Kit (GenStar) following the manufacturer's instructions. The concentrations were then measured using a NANODROP ONE (Thermo SCIENTIFIC), and purity was assessed by agarose gel electrophoresis.

### Restriction Enzymes Digestion of the Yersiniophage PhiR1‐37 Genomic DNA and dU‐DNA Fragment

Yersiniophage PhiR1‐37 genomic DNA (0.2–0.3 µg) or PCR amplified dU‐DNA fragment was subjected to the selective restriction enzymes digestion in a 20 µL reaction mixture following the manufacturer's instructions.

### dU‐DNA Fragment Digestion with SpCas9 and LbCas12a

dU‐DNA and dT‐DNA fragments (300–400 ng) were subjected to *Sp*Cas9 and *Lb*Cas12a endonuclease digestion in a 20 µL reaction mixture following the manufacturer's instructions. The sgRNA1 and sgRNA2 were used in the *Lb*Cas12a digestion assay, and sgRNA3 and sgRNA4 were used in the *Lb*Cas12a digestion assay.

### Statistical Analysis

All enzymatic assays were carried out in triplicate (*n* = 3). Data were presented as mean ± standard deviation (SD), with error bars indicating the SD. Kinetic parameters (*K_M_* and *V*_max_) were determined by nonlinear regression fitting of the Michaelis–Menten equation using GraphPad Prism 5.0.

## Conflict of Interest

The authors declare no conflict of interest.

## Author Contributions

Y.Z. and X.J. conceived the project. Y.Z., Y.W., and Y.L. designed the experiments. Y.W. and X.J. expanded the scope of the project. Y.L., J.W., Y.T., Z.Z., and Y.Z. conducted the wet and dry lab experiments. Y.W., J.T., and Y.Z. conducted the bioinformatics study. Y.L., Y.W., J.T., X.J., and Y.Z. wrote the paper.

## Supporting information



Supporting Information

## Data Availability

The data that support the findings of this study are available from the corresponding author upon reasonable request.;
