# Supplementary material for: Distinct Phage‐Encoded Enzymes for Substitution of Deoxythymidine by Deoxyuridine in Phage Genomes
Source: Adv Sci (Weinh). 2025 Sep 26;12(46):e12937. doi: 10.1002/advs.202512937 (PMC12697855; doi:10.1002/advs.202512937)
Supplement: Supplementary file 1 — Supporting Information [file ADVS-12-e12937-s001.docx]

**Supplementary Information**

**Distinct Phage Encoded Enzymes for Substitution of Deoxythymidine by Deoxyuridine in Phage Genomes**

**Yating Li^1,2,3^,** **Jason Tan^4^, Yanqin Tu^1,2,3^, Jingnan Wu^5^, Zaifang Zhang^5^, Yifeng Wei^4*^, Xinan Jiao^1,2,3*^ and Yan Zhou^1,2,3*^**

From the ^1^Jiangsu Key Laboratory of Zoonosis, Yangzhou University, Yangzhou, 225009, China.

^2^Key Laboratory of Prevention and Control of Biological Hazard Factors (Animal Origin) for Agrifood Safety and Quality, Ministry of Agriculture of China, Yangzhou University, Yangzhou, 225009, China.

^3^Jiangsu Co-Innovation Center for Prevention and Control of Important Animal Infectious Diseases and Zoonoses, Yangzhou University, Yangzhou 225009, Jiangsu Province, China.

^4^Singapore Institute of Food and Biotechnology Innovation (SIFBI), Agency for Science, Technology and Research (A*STAR), Singapore 138669, Singapore.

^5^College of Bioscience and Biotechnology, Yangzhou University, Yangzhou 225009, Jiangsu Province, China.

* To whom correspondence should be addressed: Yifeng Wei (wei_yifeng@sifbi.a-star.edu.sg); Xinan Jiao (jiao@yzu.edu.cn); Yan Zhou (yan_zhou@yzu.edu.cn)

**
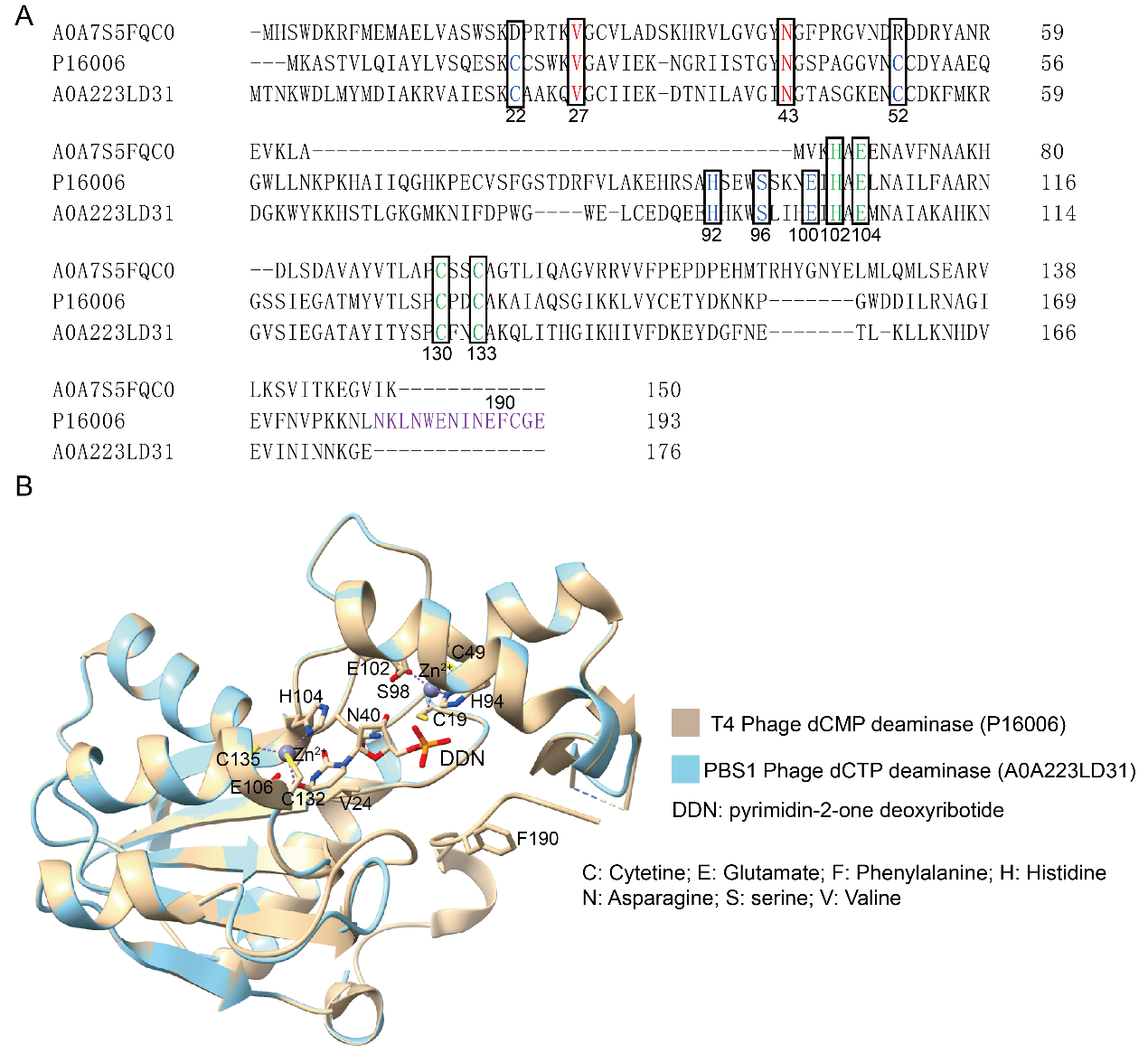
**

**Fig. S1 Sequence and structure analysis of phage cytidine deaminases**. A) Multiple sequence alignment of Dcd_PBS1 (A0A223LD31), phage T4 dCMP deaminase (1) (DCTD, P16006) and putative dCMP deaminase of phage DSS3_VP1 (A0A7S5FQC0) (2). The amino acids coordinating the catalytic metal ion (M1) are colored in green. The amino acids that coordinate the structural metal ion (M2) are colored in blue. The conserved amino acids binding the substrate nucleoside moiety (dC) are colored in red. Unlike the other two enzymes, DCTD has a C-terminal 14 amino acid extension, colored in purple. B) Structural comparison of Dcd_PBS1 and DCTD, showing the active site amino acids that bind the substrate and metal cofactors. The DCTD (P16006, PDB: 1VQ2(1)) is colored in gray, and the AlphaFold 3 (3) model of Dcd_PBS1 is colored in blue. **
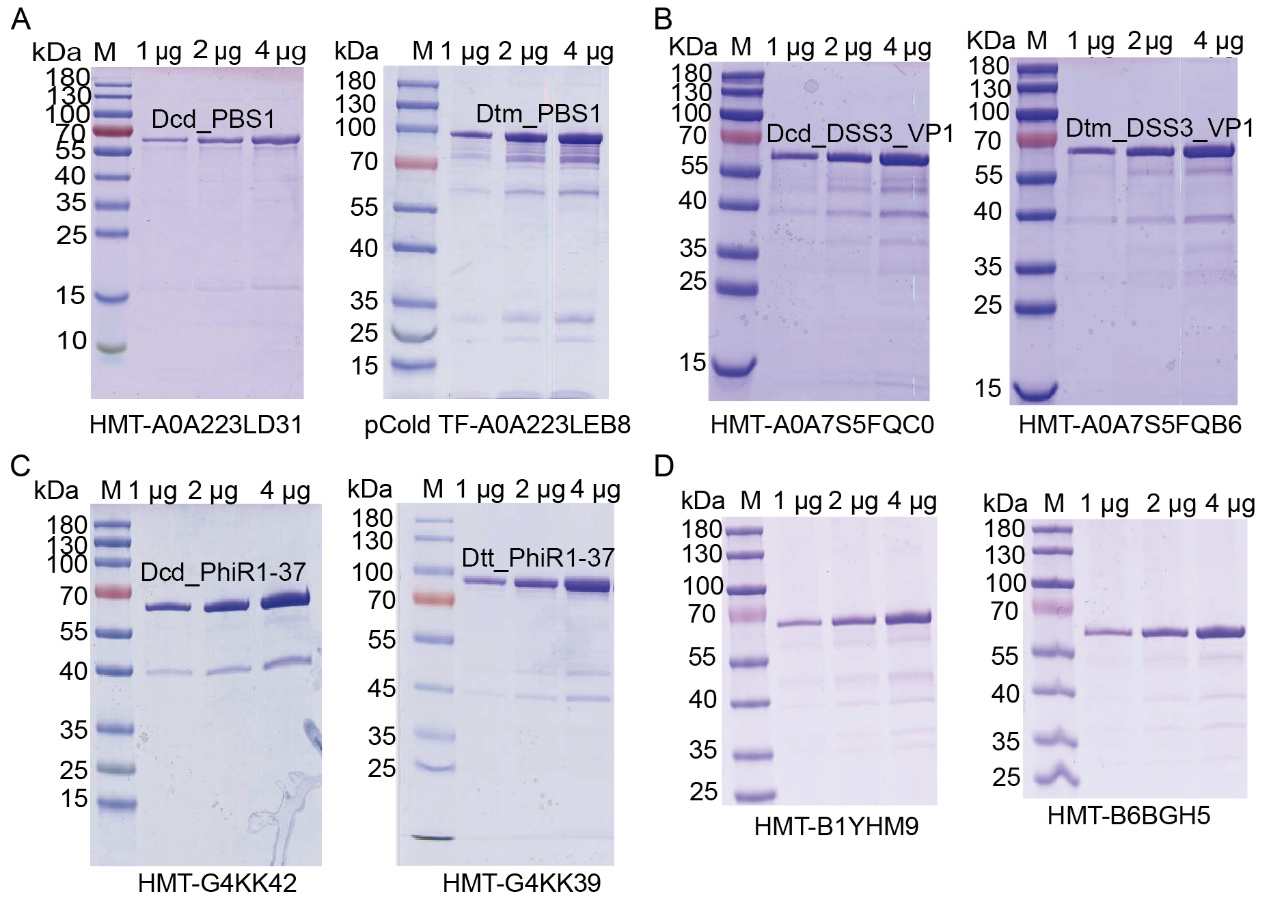
**

**Fig. S2 SDS-PAGE gel analyses of recombinant proteins purified in this study.** A-D) 10% SDS gel with lane 1, molecular weight marker (M); and lanes 2, 3 and 4 with 1, 2, 4 μg of purified proteins respectively.

**
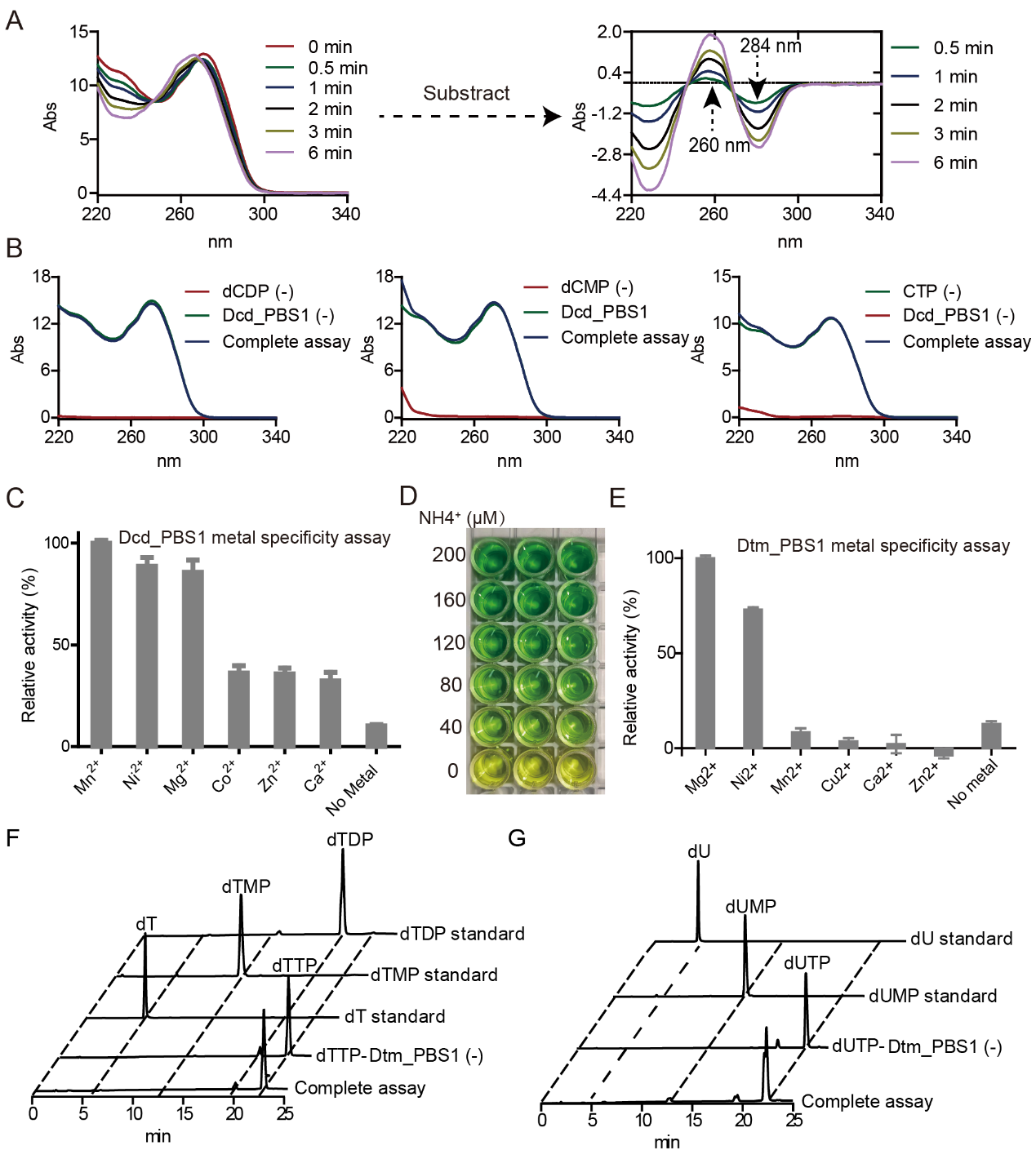
**

**Fig. S3 Dcd_PBS1 and Dtm_PBS1 enzyme assays.** A) Time-dependent UV spectra of Dcd_PBS1 assays with dCTP as substrate. Inset: UV difference spectra with the UV spectrum at time 0 subtracted from each of the spectra collected at different time points. B) Enzyme assay of Dcd_PBS1 with dCDP, dCMP or CTP as substrate**.** C) Dcd_PBS1 metal cofactor specificity assay (n=3). D) Images of microtiter plate of salicylic acid-hypochlorite colorimetric assays for NH_4_^+^ (n=3). E) Dtm_PBS1 metal cofactor specificity assay (n=3). F-G) LC-UV elution profile of Dtm_PBS1 assay with dTTP or dUTP as substrate respectively. All enzymatic assays were carried out in triplicate (n = 3). Data are presented as mean ± standard deviation (SD), with error bars indicating the SD.


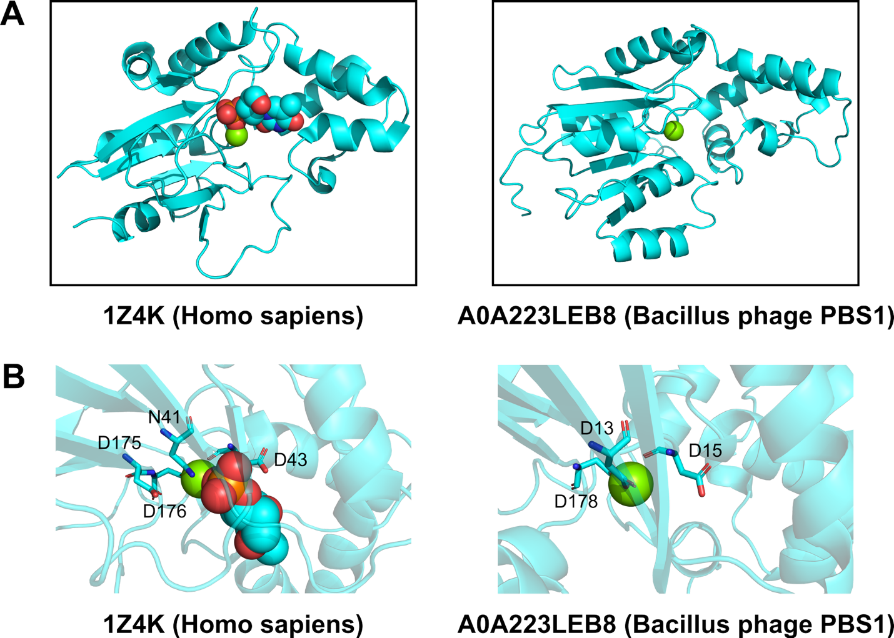


**Fig. S4. Structural comparison of human mitochondrial deoxyribonucleotidase and Bacillus phage Dtm_PBS1.** (A) Overall protein structures showing the crystal structure of human mitochondrial 5'(3')-deoxyribonucleotidase D41N mutant (PDB 1Z4K) (4) and AlphaFold3 structure of *Bacillus* phage Dtm_PBS1 (A0A223LEB8). Green spheres represent magnesium ions. The structure of 1Z4K includes bound thymidine-3'-phosphate (rendered as spheres). (B) Active site comparison showing metal-binding sites of 1Z4K and A0A223LEB8.

**
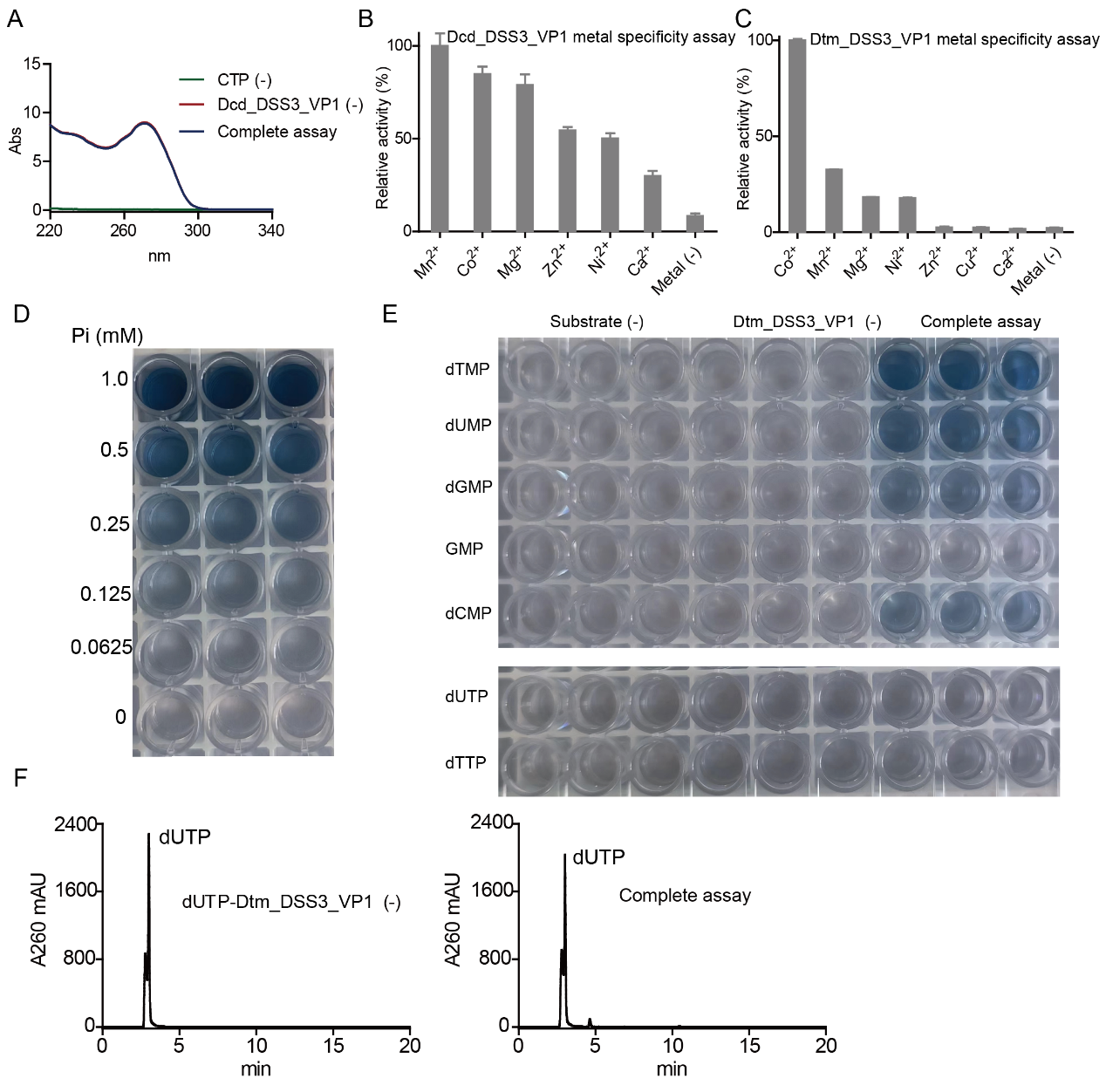
**

**Fig. S5 Dcd_DSS3_VP1 and Dtm_DSS3_VP1 enzyme assays.** A) UV spectra of Dcd_DSS3_VP1 assays with CTP as substrate**.** B) Dcd_DSS3_VP1 metal cofactor specificity assay (n=3). C) Dtm_DSS3_VP1 metal cofactor specificity assay (n=3). D) Images of microtiter plate of phosphomolybdate colorimetric assays for phosphate (Pi, 1.0-0 mM) (n=3). E) The substrate specificity assay for Dtm_DSS3_VP1 (n=3). F) LC-UV elution profile of Dtm_DSS3_VP1 assay with dUTP as substrate. All enzymatic assays were carried out in triplicate (n = 3). Data are presented as mean ± standard deviation (SD), with error bars indicating the SD.

**
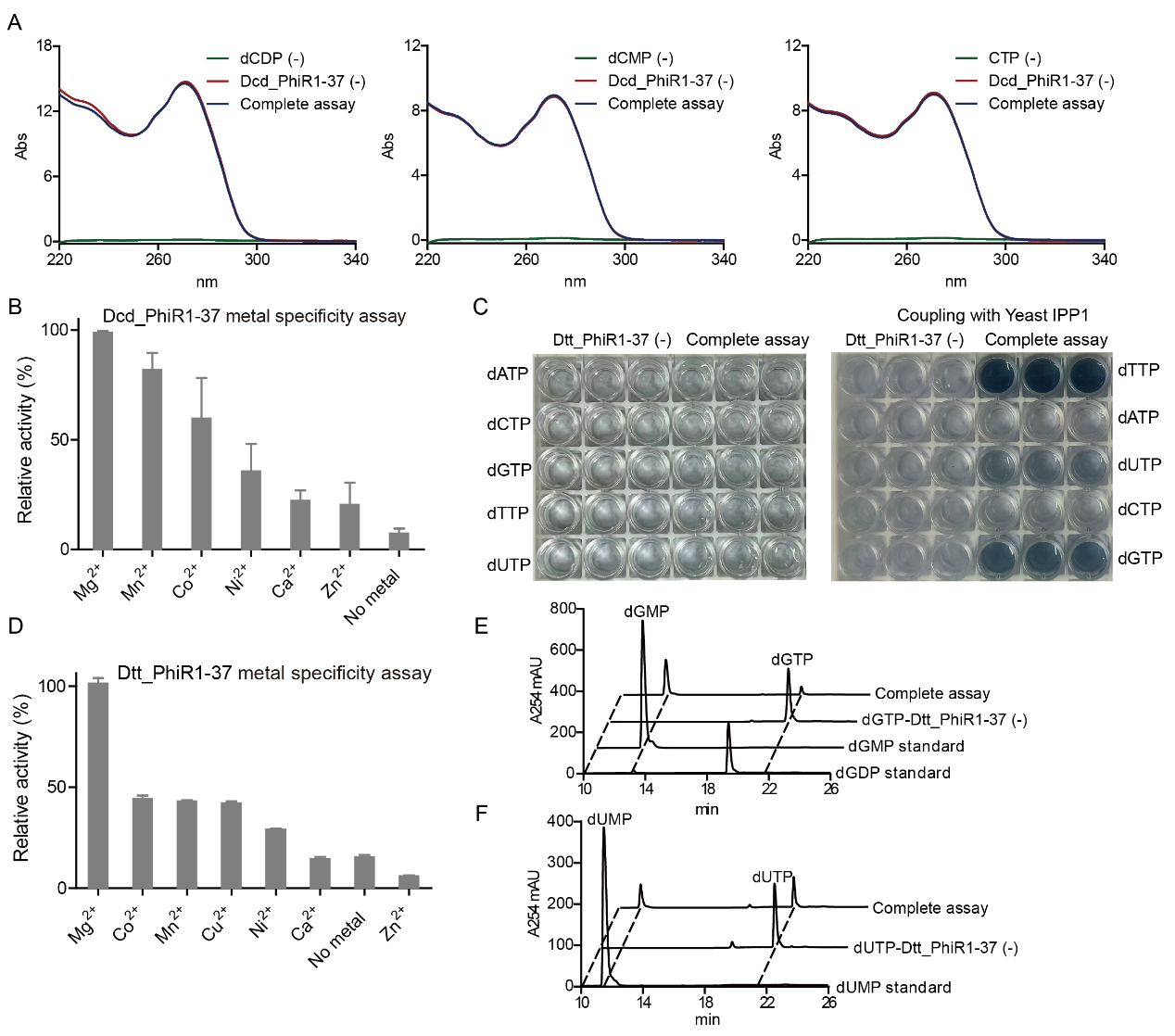
**

**Fig. S6 Dcd_PhiR1-37 and Dtt_PhiR1-37 enzyme assays.** A) UV spectra of Dcd_PhiR1-37 assays with dCDP, dCMP or CTP as substrate**.** B) Dcd_PhiR1-37 metal cofactor specificity assay (n=3). C) The substrate specificity assay for Dtt_PhiR1-37 (n=3). D) Dtt_PhiR1-37 metal cofactor specificity assay (n=3). E*-*F) LC-UV elution profile of Dtt_PhiR1-37 assay with dGTP or dUTP as substrate respectively. All enzymatic assays were carried out in triplicate (n = 3). Data are presented as mean ± standard deviation (SD), with error bars indicating the SD.

**
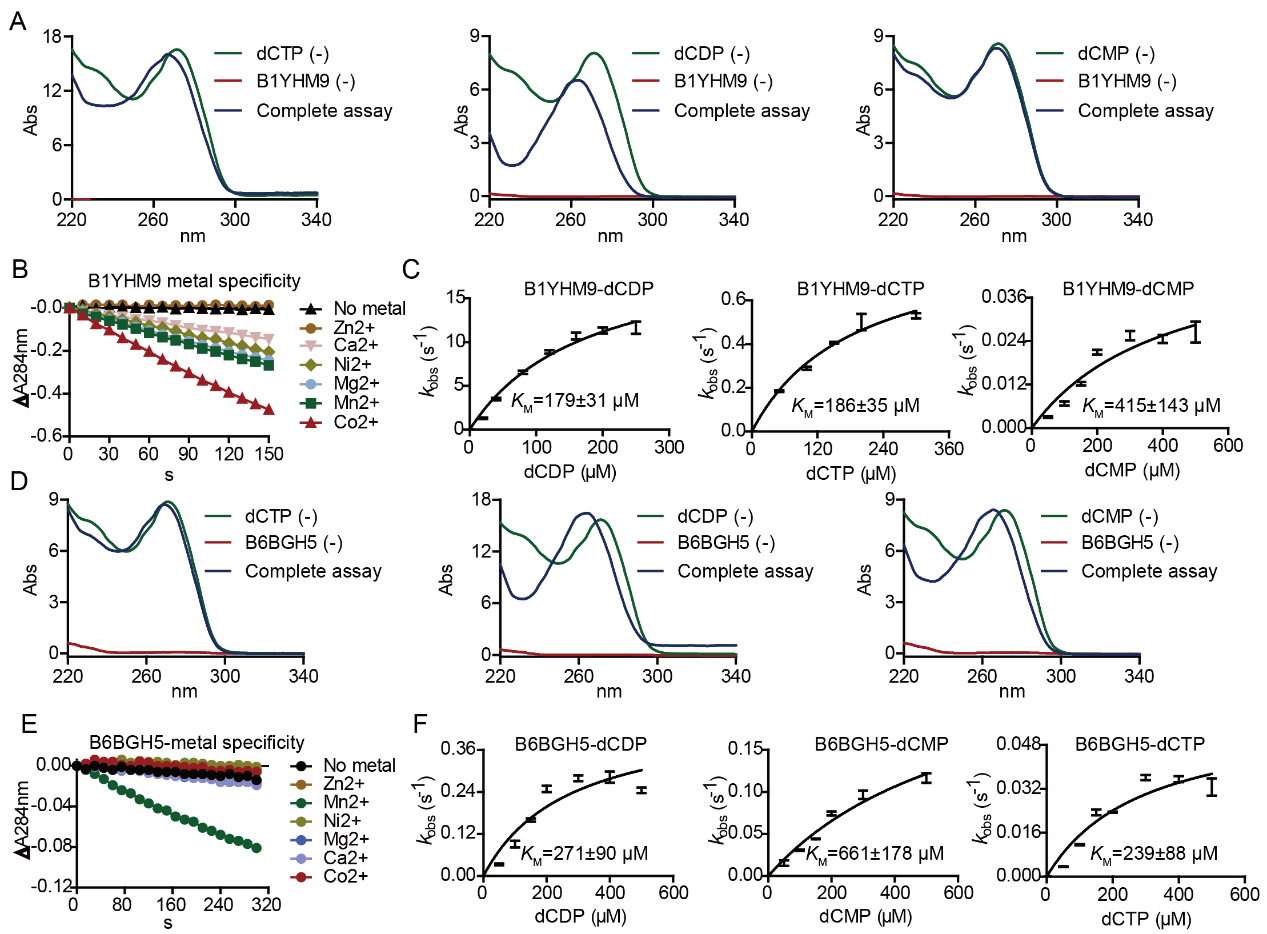
**

**Fig. S7 Bacterium dCDP deaminases activity assays.** A) Substrates specificity assay of *E. sibiricum* dCDP deaminase (B1YHM9). B) *E. sibiricum* dCDP deaminase (B1YHM9) metal cofactor specificity assay. C) Michaelis–Menten kinetics of B1YHM9 varying the concentrations of dCDP, dCTP or dCMP respectively (n=3). D) Substrates specificity assay of *S.* *gotlandica* dCDP deaminase (B6BGH5). E) *S.* *gotlandica* dCDP deaminase (B6BGH5) metal cofactor specificity assay. F) Michaelis–Menten kinetics of B6BGH5 varying the concentrations of dCDP, dCTP or dCMP respectively (n=3). All enzymatic assays were carried out in triplicate (n = 3). Data are presented as mean ± standard deviation (SD), with error bars indicating the SD.


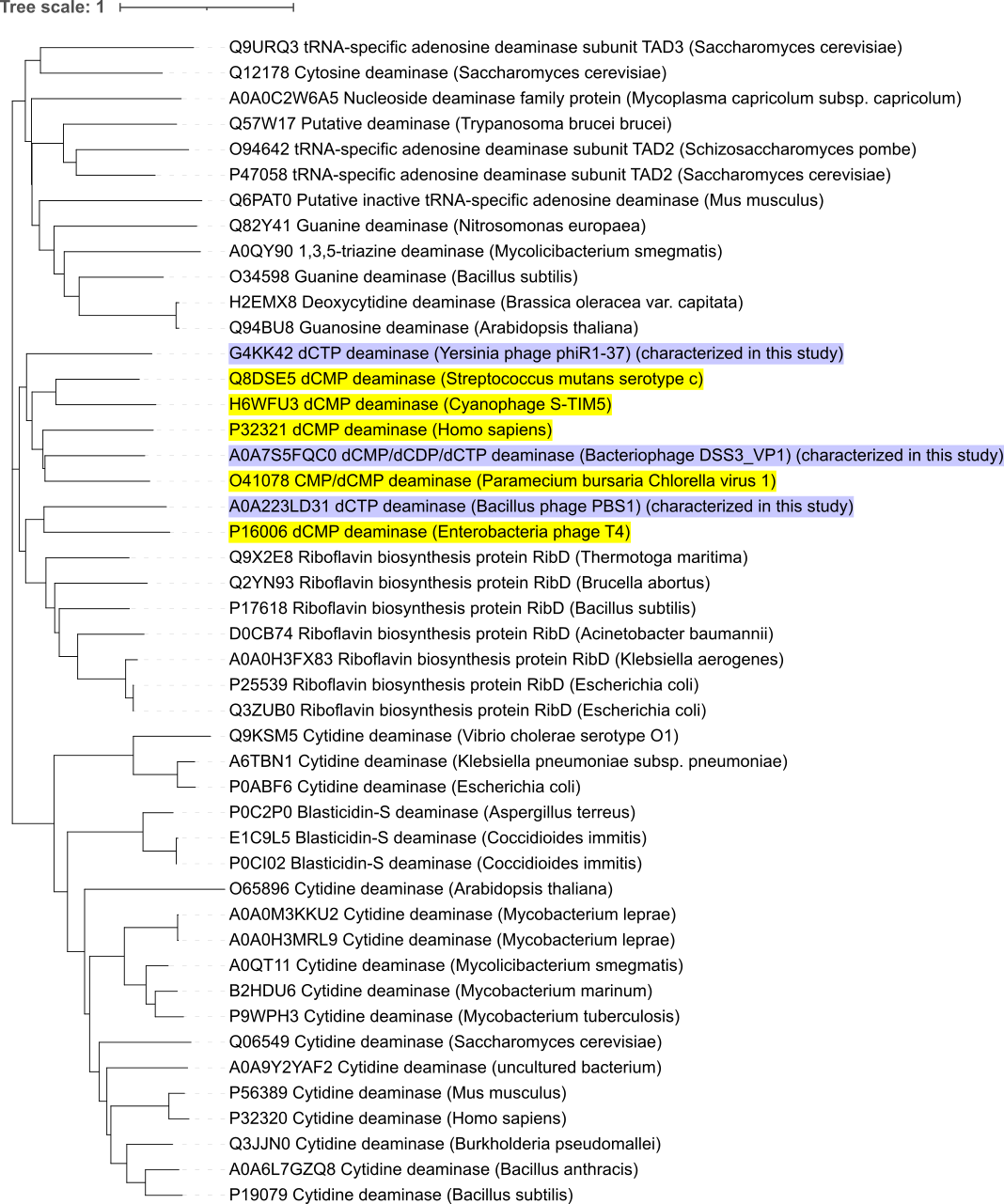


**Fig. S8 Phylogenetic tree of dCTP deaminase family enzymes**. A phylogenetic tree was constructed using 43 crystallographically characterized members of the cytidine deaminase family (PF00383) and three phage deaminases (A0A223LD31, A0A7S5FQC0 and G4KK42) characterized in this study. Multiple sequence alignment was performed using MUSCLE software, followed by construction of a neighbor-joining phylogenetic tree using MEGA software. The tree was rooted at the midpoint and visualized using iTOL web-based software. The CMP/dCMP deaminases are highlighted in yellow, while the three phage deaminases characterized in this study are highlighted in blue.


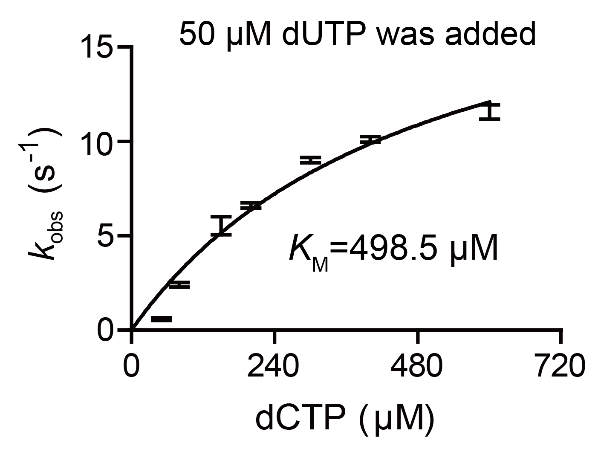


**Fig. S9 The allosteric regulation properties of Dcd_PBS1**. Michaelis–Menten kinetics of Dcd_PBS1 with 50 μM dUTP varying the concentrations of dCTP (n=3). The enzymatic assay was carried out in triplicate (n = 3). Data are presented as mean ± standard deviation (SD), with error bars indicating the SD.

**References**

1. Almog, R., Maley, F., Maley, G. F., Maccoll, R., and Van Roey, P. (2004) Three-dimensional structure of the R115E mutant of T4-bacteriophage 2'-deoxycytidylate deaminase. *Biochemistry* **43**, 13715-13723

2. Rihtman, B., Puxty, R. J., Hapeshi, A., Lee, Y. J., Zhan, Y., Michniewski, S., Waterfield, N. R., Chen, F., Weigele, P., Millard, A. D., Scanlan, D. J., and Chen, Y. (2021) A new family of globally distributed lytic roseophages with unusual deoxythymidine to deoxyuridine substitution. *Curr Biol* **31**, 3199-3206 e3194

3. Abramson, J., Adler, J., Dunger, J., Evans, R., Green, T., Pritzel, A., Ronneberger, O., Willmore, L., Ballard, A. J., Bambrick, J., Bodenstein, S. W., Evans, D. A., Hung, C. C., O'Neill, M., Reiman, D., Tunyasuvunakool, K., Wu, Z., Zemgulyte, A., Arvaniti, E., Beattie, C., Bertolli, O., Bridgland, A., Cherepanov, A., Congreve, M., Cowen-Rivers, A. I., Cowie, A., Figurnov, M., Fuchs, F. B., Gladman, H., Jain, R., Khan, Y. A., Low, C. M. R., Perlin, K., Potapenko, A., Savy, P., Singh, S., Stecula, A., Thillaisundaram, A., Tong, C., Yakneen, S., Zhong, E. D., Zielinski, M., Zidek, A., Bapst, V., Kohli, P., Jaderberg, M., Hassabis, D., and Jumper, J. M. (2024) Accurate structure prediction of biomolecular interactions with AlphaFold 3. *Nature* **630**, 493-500

4. Wallden, K., Ruzzenente, B., Rinaldo-Matthis, A., Bianchi, V., and Nordlund, P. (2005) Structural basis for substrate specificity of the human mitochondrial deoxyribonucleotidase. *Structure* **13**, 1081-1088
